# DNMT1 Regulates Testosterone Synthesis in Aged Rat Leydig Cells via FTO‐Mediated HSP90AA1 m^6^A Levels

**DOI:** 10.1111/acel.70760

**Published:** 2026-10-12

**Authors:** Yaqi Li, Yulei Duan, Zihang Tian, Yibing Gao, Yufei Qiao, Shuang Zhao, Wenyan Li, Zheng Gao, Siyun Niu

**Affiliations:** ^1^ School of Basic Medical Sciences Hebei University Baoding PR China

**Keywords:** aging, AKT signaling, DNMT1, FTO, HSP90AA1, Leydig cells, testosterone

## Abstract

Testosterone levels decline progressively with age from the third decade of life, impairing spermatogenesis and contributing to age‐related disorders, underscoring the importance of enhancing endogenous testosterone production. DNA methyltransferase 1 (DNMT1) promotes testosterone synthesis in Leydig cells (LCs), yet the underlying mechanism remains unclear. Here, we found that DNMT1 expression is downregulated in aging rat testes and senescent LCs in vitro, correlating with reduced testosterone levels. Gain‐ and loss‐of‐function experiments confirmed that DNMT1 positively regulates testosterone synthesis and alleviates LC senescence, and that DNMT1 overexpression reverses the senescent phenotype and restores testosterone production. Mechanistically, RNA‐seq of DNMT1‐knockdown LCs identified FTO as a downstream target; DNMT1 suppresses FTO expression by promoting *Fto* promoter methylation. catRAPID prediction and RIP‐qPCR validated *Hsp90aa1* mRNA as a direct FTO substrate; FTO regulates HSP90AA1 expression through m^6^A modification, with DNMT1 indirectly modulating HSP90AA1 via FTO. Using HSP90AA1 overexpression, pharmacological inhibition, and rescue experiments, we confirmed that the DNMT1–FTO axis inhibits LC apoptosis through HSP90AA1‐mediated AKT activation, thereby promoting testosterone synthesis. Expression patterns of this axis in aging rat testes and senescent LCs further corroborated its involvement in age‐related testosterone dysregulation. In vivo AAV‐mediated testicular DNMT1 knockdown recapitulated these findings. Collectively, our study elucidates the physiological role of DNMT1 in age‐related testosterone decline and delineates the mechanism by which DNMT1 governs LC senescence and steroidogenic function, providing a potential therapeutic target for reproductive system aging.

## Graphical abstract

During Leydig cell senescence, decreased DNMT1 triggers *Fto* promoter hypomethylation and upregulates FTO. Elevated FTO reduces m^6^A modification of *Hsp90aa1*, lowering HSP90AA1 to inhibit AKT signaling, facilitate apoptosis and reduce testosterone synthesis.

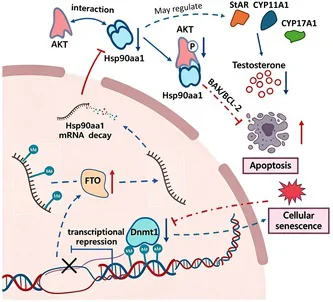

## 1 Introduction

Testosterone serves as the primary driver of male reproductive function. It not only sustains spermatogenesis but also participates in the modulation of multiple systemic processes, with its deficiency closely associated with immune dysfunction, decreased bone density, cardiovascular diseases, and sleep disorders (Handelsman and Wittert 2024; Kaufman and Lapauw 2020; Ketchem et al. 2023; Liu and Reddy 2022; Moreau et al. 2020; Tenuta et al. 2025). Therefore, maintaining adequate endogenous testosterone levels is essential for male physiological homeostasis. Epigenetic alterations represent one of the hallmarks of aging. Programmed changes in the epigenome are regarded as both key indicators and drivers of the aging process because of their central role in regulating gene expression (López‐Otín et al. 2023; Wu et al. 2024). Among various epigenetic modifications, DNA methylation is one of the most stable and well‐characterized, playing critical roles in spermatogenesis, testosterone synthesis, and cellular senescence (Field et al. 2018;   Li, Jiang, et al. 2023; Maroto et al. 2025; Xu et al. 2025). DNA methyltransferase 1 (DNMT1) is a central enzyme responsible for maintaining genomic methylation homeostasis, and its dysregulated expression is strongly associated with reproductive disorders and aging‐related phenotypes. In the testis, DNMT1 preserves normal proliferation and differentiation of spermatogenic cells by stabilizing DNA methylation patterns (Takada et al. 2021). With advancing age, DNMT1 activity gradually decreases, which disrupts genomic methylation patterns and dysregulates the expression of downstream functional genes (Guo et al. 2025; Li et al. 2010). Notably, a multi‐tissue transcriptomic analysis revealed that DNMT1 is abundantly and preferentially expressed in the testis compared with 31 other human tissues (Dezso et al. 2008), suggesting that DNMT1 may function as a core methyltransferase governing testicular development and functional stability. Leydig cells (LCs) are the primary cellular source of testicular testosterone synthesis, and their functional status directly determines circulating testosterone levels. Nevertheless, the precise molecular mechanism by which DNMT1 modulates testosterone synthesis in LCs remains largely unclarified. Further exploration of its regulatory mechanism will provide novel theoretical basis and potential intervention targets for improving endogenous testosterone synthesis and delaying male reproductive aging.

As a major type of post‐transcriptional modification, N^6^‐methyladenosine (m^6^A) plays a crucial regulatory role in mammalian spermatogenesis and the maintenance of reproductive function. Recent studies indicate crosstalk between DNA methylation and m^6^A in cancer, liver fibrosis, and renal inflammation (Dong et al. 2024; Li, Zheng, et al. 2023; Yu et al. 2021; Zhang et al. 2020); however, the interaction between these two epigenetic layers has rarely been investigated in the context of reproductive aging. FTO, a well‐characterized m^6^A demethylase, is ubiquitously expressed in mammalian tissues and participates in a broad spectrum of physiological and pathological processes (Huang et al. 2022; Lin et al. 2023; Qiao et al. 2024; Zou et al. 2024). Previous studies have reported that FTO expression is significantly upregulated in multiple senescent organs of aged mice, including the heart, brain, lung, and kidney (Krejčí et al. 2023), suggesting a broad involvement of FTO in organismal senescence. In the testis, FTO modulates spermatogenesis by regulating the m^6^A levels of key genes in Sertoli cells and spermatogenic cells (Zhang et al. 2025). FTO dysfunction may directly lead to spermatogenic abnormalities (Wu et al. 2023). Accordingly, we hypothesized that the regulatory effect of DNMT1 on age‐related testosterone decline may be mediated through DNMT1–FTO epigenetic crosstalk, which modulates the expression of downstream target genes in senescent LCs.

HSP90AA1 encodes a vital molecular chaperone that participates in protein folding, cellular stress response, proliferation, apoptosis, and signal transduction (Chen et al. 2023; Wang et al. 2025; Yan et al. 2024; Yan et al. 2025). As a critical chaperone of AKT, HSP90AA1 exerts anti‐apoptotic effects by stabilizing and activating AKT signaling (Zhang et al. 2023). Based on these findings, we proposed that the DNMT1–FTO axis suppresses LC apoptosis and facilitates testosterone synthesis via HSP90AA1‐dependent AKT activation. The present study explores the mechanism by which DNMT1 elevates endogenous testosterone levels. We reveal that DNMT1 sustains high DNA methylation of FTO to silence its expression. Reduced FTO expression elevates the m^6^A modification of HSP90AA1 and enhances HSP90AA1 abundance; consequently, upregulated HSP90AA1 activates AKT signaling to suppress LC apoptosis and promote testosterone synthesis. Collectively, this study defines the role of the DNMT1–FTO‐mediated HSP90AA1–AKT pathway in endogenous testosterone production, and the DNMT1–FTO axis represents a potential therapeutic target to enhance endogenous testosterone secretion.

## 2 Materials and Methods

### 2.1 Experimental Animal Model

Forty male Wistar rats (2 months old, weighing 350–390 g) were obtained from Beijing SPF Biotechnology Co. Ltd. (Beijing, China). Animals were housed under specific pathogen‐free (SPF) conditions (22°C ± 0.2°C, 12‐h light/dark cycle) with ad libitum access to food and water. After 1 week of acclimatization, rats were randomly assigned using a computer‐generated random number table to four experimental groups (2‐, 6‐, 12‐, and 18‐month‐old; *n* = 10 per group), and maintained under standard husbandry conditions until the designated age for tissue collection.

For in vivo gene knockdown, adeno‐associated virus serotype 9 (AAV9) carrying a construct targeting *Dnmt1* was administered via local intratesticular injection to achieve specific knockdown of the *Dnmt1* gene in rat testicular tissues (Shanghai GeneChem Co. Ltd., China; titer: 1.87 × 10^13^ vg/mL) (Wang et al. 2019; Xia et al. 2022; Zhang et al. 2024). To determine the optimal viral titer, three graded doses—8 × 10^9^, 4 × 10^10^, and 2 × 10^11^ vg—were tested (*n* = 3 per dose). Following titer optimization, the formal experiment was conducted with five rats per group. Two‐month‐old rats were anesthetized with pentobarbital sodium (50 mg/kg, i.p.), and a 2 cm scrotal incision was made to expose the testes. A sterile insulin syringe was used to deliver the AAV9 suspension (diluted in PBS) via multi‐site intratesticular injection. The tunica albuginea was punctured, and the virus was injected at four sites (superior, inferior, left, and right) per testis, with 50 μL per site, totaling 200 μL per rat. Following injection, the incision was closed with interrupted absorbable sutures, and each rat received an intramuscular injection of 100,000 units of penicillin. The wound was disinfected daily with iodophor swabs for the first 5 days post‐surgery. Testicular tissues were harvested 3 weeks after viral injection for subsequent analyses.

All animal procedures were performed in accordance with the institutional guidelines for laboratory animal care and approved by the Animal Ethics and Experimental Innovation Committee (approval no. AEEI‐2016‐143; October 17, 2016).

### 2.2 Isolation, Culture, and Identification of Primary Leydig Cells

Primary Leydig cells (LCs) were isolated from male Wistar rats at postnatal days 15–20, 2 months, or 18 months of age following previously described protocols with minor modifications (Ji et al. 2022; Liang et al. 2021; Zhou et al. 2016). Rats were euthanized by cervical dislocation, and bilateral testes were harvested, rinsed twice in pre‐cooled phosphate‐buffered saline (PBS), and transferred to twice the tissue volume of collagenase I solution (1 mg/mL; C8140; Solarbio) in PBS. Testes were digested in a 37°C water bath for 15 min with intermittent shaking (3–5 times, 10 s each) until the tissue became flocculent and dissociated. Digestion was terminated by adding an equal volume of complete medium, consisting of DMEM/F12 (L104‐500; BDBio) supplemented with 10% fetal bovine serum (FBS; S9020; Solarbio) and 1% penicillin–streptomycin (PS; 450‐201‐EL; WISENT). The suspension was gently triturated, allowed to stand for 5 min, and then filtered through a 300‐mesh stainless steel sieve. The filtrate was centrifuged at 1500 rpm for 10 min. The supernatant was discarded, and the cell pellet was washed once with PBS, resuspended in complete medium, and seeded into culture dishes. Cells were incubated at 37°C in a humidified atmosphere containing 5% CO_2_. After 1 h, non‐adherent cells were removed, and adherent LCs were washed twice with PBS and cultured in fresh complete medium.

For 3β‐hydroxysteroid dehydrogenase (3β‐HSD) staining (Jiang et al. 2015; Li et al. 2019), cells were cultured for 48 h until reaching 80%–90% confluence. Stock solutions were prepared as follows: Nitro Blue Tetrazolium (NBT; B494228; Aladdin) was dissolved in DMSO (ID9011; Solarbio) at a final concentration of 5 mg/mL; trans‐Dehydroandrosterone (DHEA; D106380; Aladdin) was dissolved in DMSO at 10 mM; β‐Nicotinamide adenine dinucleotide (β‐NAD; N1492518; Aladdin) was dissolved in PBS at 100 mg/mL; and Nicotinamide (NAM; N108087; Aladdin) was dissolved in PBS at 50 mg/mL. The working solution (1 mL) was freshly prepared by mixing 30 μL NBT stock, 55.5 μL DHEA stock, 3.6 μL β‐NAD stock, and 3.2 μL NAM stock in 907.7 μL PBS. Cells were incubated with the working solution overnight at 37°C and examined under a microscope the following day. LCs were identified by the presence of blue‐purple formazan precipitates in the cytoplasm, indicative of 3β‐HSD activity.

### 2.3 Senescence‐Associated β‐Galactosidase (SA‐β‐Gal) Staining

SA‐β‐gal staining was performed using a commercial kit (C0602; Beyotime) to identify senescent cells. The working solution was freshly prepared by mixing 10 μL staining solution A, 10 μL staining solution B, 930 μL staining solution C, and 50 μL X‐Gal (provided in the kit) in a sterile tube. Cells were washed three times with PBS (3 min each), fixed with 1 mL fixative solution per well for 20 min at room temperature, and washed three more times with PBS (3 min each). The freshly prepared working solution (1 mL) was then added to each well, and cells were incubated at 37°C for 16–18 h in a dry incubator (avoiding CO_2_). After washing with PBS, cells were examined under a light microscope. Five random fields per well were captured, and cells with blue cytoplasmic staining were counted as SA‐β‐gal‐positive. The percentage of positive cells was calculated relative to the total number of cells per field.

### 2.4 Cell Viability Assay

In a 96‐well plate, seed LCs at a density of 8000 cells per well. Six replicate wells should be used for each concentration gradient of the HSP90AA1 inhibitor 17‐AAG, which should be set at 0, 0.5, 1, and 2 μmol/L. Three time intervals should be incubated: 24, 48, and 72 h. Each well should receive 10 μL of CCK‐8 (GK10001; GLPBIO) reagent after incubation. For 2 h, incubate at 37°C. Utilizing a microplate reader, determine cell viability by measuring absorbance at 450 nm.

### 2.5 Methylation‐Specific PCR (MSP)

Genomic DNA was extracted from frozen testicular tissues and primary Leydig cells using the TIANamp Genomic DNA Kit (DP302‐02; TIANGEN) according to the manufacturer's instructions. DNA concentration and purity were assessed using a Nanodrop One spectrophotometer, and all samples were diluted to a uniform concentration of 25 ng/μL. Bisulfite conversion of genomic DNA was performed using the EZ DNA Methylation‐Gold Kit (D5006; ZYMO RESEARCH). Briefly, genomic DNA was mixed with CT Conversion Reagent and subjected to denaturation and bisulfite conversion in a thermal cycler under the following conditions: 98°C for 10 min, 64°C for 2.5 h, and 4°C for 5 min. After conversion, the samples were loaded onto spin columns, followed by sequential binding, washing, desulfonation, and elution steps according to the manufacturer's protocol. The modified DNA was then eluted and stored for further analysis.

CpG islands in the *Fto* and *Hsp90aa1* promoters were predicted using the UCSC Genome Browser and MethPrimer 2.0 (criteria: length ≥ 200 bp, GC content > 50%, observed/expected CpG ratio > 0.6). For *Fto*, the primers target a promoter CpG island at chr19:15,691,734–15,692,493 (rn7; 760 bp, GC content 59.3%, observed/expected CpG 0.83, 54 CpG sites), with the M/U amplicons spanning chr19:15,692,248–15,692,367 (120 bp, 11 CpG dinucleotides). For *Hsp90aa1*, the primers target a promoter CpG island at chr6:129,707,250–129,708,600 (rn7; 1351 bp, GC content 66.1%, observed/expected CpG 0.88, 129 CpG sites), with the M/U amplicons spanning chr6:129,708,407–129,708,490 (84 bp, 8 CpG dinucleotides). The primer sequences were aligned against the bisulfite‐converted rat genome (rn7) using the UCSC Genome Browser, confirming unique annealing within the target CpG island regions.

MSP primers were synthesized by Sangon Biotech (Shanghai) Co. Ltd. (Shanghai, China). Primer sequences are listed in Table 1. MSP amplification was carried out using the ZymoTaq PreMix Kit (E2003; ZYMO RESEARCH). The 20 μL reaction mixture contained 10 μL PCR premix, 1 μL forward primer (10 μM), 1 μL reverse primer (10 μM), 1 μL bisulfite‐converted DNA template, and 7 μL nuclease‐free water. The PCR cycling conditions were as follows: initial denaturation at 95°C for 10 min; 40 cycles of denaturation at 94°C for 30 s, annealing at optimized temperatures (see Table 1) for 30 s, and extension at 72°C for 30 s; with a final extension at 72°C for 10 min.

**TABLE 1:** Primers used in this study.

Gene	Primer sequences (5′ → 3′)	Annealing temperature (°C)
For methylation‐specific PCR		
*Fto*	M‐F: GTTTAGAGGAGTACGGGAAAATAGC	52
	M‐R: TACGTTAGTTTTGTTGGTTGATTG	
	U‐F: GGTTTAGAGGAGTATGGGAAAATAGT	
	U‐R: TATGTTAGTTTTGTTGGTTGATTGG	
*Hsp90aa1*	M‐F: CGTAATAAGTAGAATTCGGGTTCGG	51
	M‐R: TCTTCGAACTCCCATTAAAAACGTA	
	U‐F: TGTAATAAGTAGAATTTGGGTTTGG	
	U‐R: TCTTCAAACTCCCATTAAAAACATA	
For quantitative real‐time PCR		
*Dnmt1*	F: CCACTGTTCCTCCTTCTGCCATC	60
	R: TCATCGTCCTTAGCGTCGTCGTA	
*Fto*	F: TTCTGTCTGCCGTCCTGATCCC	60
	R: GTCTGGTTTCTGCTGTGCTGGTAG	
*Hsp90aa1*	F: CAAGCAAGACCGAACCCTCACT	60
	R: CAGGCGTACTGCTCGTCATCAT	
*Star*	F: GGTTCTCAACTGGAAGCAACACTC	60
	R: CACACCTGGCACCACCTTACTT	
*Cyp11a1*	F: TTGCTGCTTGATGCGTCTGTGTAA	60
	R: ACTCTTCTCCTGCGAGGGTCCTA	
*Cyp17a1*	F: CTATCCGAGAAGTGCTGCGTATCA	60
	R: TCTGCGTGGGTGTAATGAGATGG	
*Tnf*	F: TCCGCTTGGTGGTTTGCTACTA	60
	R: GTGCCTCAGCCTCTTCTCATTCCT	
*Il1b*	F: TCGTGCTGTCTGACCCATGTGAG	60
	R: TGTCCCGACCATTGCTGTTTCCT	
*Il6*	F: ACTTCCAGCCAGTTGCCTTCTTG	60
	R: TGGTCTGTTGTGGGTGGTATCCT	
*Actb*	F: TCAGGTCATCACTATCGGCAAT	60
	R: AAAGAAAGGGTGTAAAACGCA	
For MeRIP‐qPCR		
*Hsp90aa1*	F: TGTGCTCTGGAGGACTGTTGA	60
	R: TTGTCACGTTCCTTCTCCACCT	
For ChIP		
*Fto*	F: CGCAGAATCCCAGATGCCC	62
	R: AGGCGGGAGACAGTCGTTG	

PCR products were resolved by electrophoresis on 2% agarose gels at 120 V for 40 min, and band images were captured using a gel imaging system. The intensities of methylated and unmethylated bands were quantified using ImageJ software. The promoter methylation level was calculated as the ratio of the methylated band intensity to the sum of methylated and unmethylated band intensities, expressed as a percentage: methylation (%) = [*M*/(*M* + *U*)] × 100%, where *M* represents the methylated band intensity and *U* represents the unmethylated band intensity.

### 2.6 Real‐Time Quantitative PCR (RT‐qPCR)

Total RNA from testicular tissue and Leydig cells was extracted using the TRIzol method. Briefly, samples were lysed, followed by chloroform extraction, isopropanol precipitation, and 75% ethanol washing. The RNA pellet was dissolved in diethyl pyrocarbonate (DEPC)‐treated water. RNA concentration and purity were assessed using a Nanodrop One spectrophotometer. First‐strand cDNA was synthesized from 1 μg of total RNA using the PrimeScript RT Reagent Kit (RR047A; TaKaRa) under the following conditions: 37°C for 15 min for reverse transcription, 85°C for 5 s for enzyme inactivation, followed by cooling to 4°C. Quantitative PCR was performed using TB Green Ex Taq II (RR820A; TaKaRa) on a LightCycler 96 real‐time PCR system (Roche Diagnostics, Switzerland). The 20 μL reaction mixture contained 10 μL TB Green Premix, 0.8 μL forward primer (10 μM), 0.8 μL reverse primer (10 μM), 2 μL cDNA template, and 6.4 μL nuclease‐free water. Each sample was run in triplicate. The thermal cycling protocol was as follows: initial denaturation at 95°C for 30 s; 40 cycles of 95°C for 5 s and 60°C for 30 s; followed by melting curve analysis to verify amplicon specificity. *Actb* (encoding β‐actin) was used as the internal reference gene. Relative mRNA expression levels were calculated using the $2^{-∆∆C_{t}}$ method. All primer sequences are provided in Table 1.

### 2.7 MeRIP‐qPCR

Total RNA was extracted using the EpiQuik CUT&RUN m^6^A RNA Enrichment (MeRIP) Kit (P‐9018‐24; EPIGENTEK), followed by RNA fragmentation and input aliquoting. Briefly, total RNA was fragmented by incubation with RNA fragmentation reagents provided in the kit. m^6^A‐containing RNA fragments were then immunoprecipitated using magnetic beads conjugated with an anti‐m^6^A antibody. After washing and elution, the enriched RNA fractions (IP) and input controls were collected. Based on the m^6^A peak positions identified from our previous MeRIP‐seq data, the sequences of modified regions on *Hsp90aa1* transcripts were retrieved via the UCSC Genome Browser, and gene‐specific primers were designed. Primer sequences are listed in Table 1. Quantitative real‐time PCR was performed using the LightCycler 96 real‐time PCR system (Roche Diagnostics, Switzerland) with TB Green Premix Ex Taq II (RR820A; TaKaRa). The relative enrichment of m^6^A modification was calculated as the percentage of input using the following formula:

$$\left(\mathrm{IP}/\text{Input}\right)\%=2^{\left(C_{t}\left(\mathrm{IP}\right)-C_{t}\left(\text{Input}\right)\right)-\log\left(0.025,2\right)}\times 100.$$

### 2.8 RIP‐qPCR

RNA immunoprecipitation (RIP) was performed using the Magna RIP RNA‐Binding Protein Immunoprecipitation Kit (17‐700; Merck) according to the manufacturer's protocol. Briefly, tissues or cells were fully lysed in RIP lysis buffer, and the supernatant was collected for immunoprecipitation. The RNA‐binding protein of interest was immunoprecipitated using a specific anti‐FTO antibody, whereas normal rabbit IgG was used as a negative control. After washing, the co‐precipitated RNA was purified, treated with DNase I to remove genomic DNA contamination, and eluted in nuclease‐free water. The enriched RNA fragments were then reverse‐transcribed into cDNA, and quantitative real‐time PCR was performed to detect the target RNA molecules bound to the protein.

### 2.9 Chromatin Immunoprecipitation (ChIP)

ChIP assays were performed using a commercial kit (JKR23002A; Wuhan Jingkai Rui) according to the manufacturer's instructions. Briefly, 1 × 10^7^ Leydig cells were crosslinked with 1 mL of 1% formaldehyde in PBS for 5 min at room temperature with rotation, and glycine was subsequently added to terminate the crosslinking reaction. After cell lysis, chromatin was sheared in an ice‐water bath using a probe sonicator (SCIENTZ‐IID; Scientz) at 150 W, with 5 s on and 2 s off pulses for a total of 15 min. Two percent of the sheared chromatin was retained as the Input sample. The remaining lysate was divided equally into two aliquots and incubated overnight at 4°C with rotation with either rabbit anti‐DNMT1 monoclonal antibody or control rabbit IgG. After sequential washing, elution, and reverse crosslinking, the purified DNA was subjected to qPCR analysis on a LightCycler 96 real‐time PCR system (Roche Diagnostics, Switzerland) using TB Green Premix Ex Taq II (RR820A; TaKaRa) with primers targeting the CpG island region within the Fto promoter. Primer sequences are listed in Table 1. Enrichment levels were calculated using the $2^{-∆∆C_{t}}$ method and presented as fold enrichment relative to IgG and as percentage of Input. All ChIP experiments were performed in three independent replicates.

### 2.10 Cell Transfection Experiments

Lentiviruses for DNMT1 knockdown (shDNMT1) were purchased from VectorBuilder Biotechnology Co. Ltd. Lentiviruses for FTO overexpression (LVFTO) and FTO knockdown (shFTO) were purchased from GenePharma Co. Ltd. Lentiviruses for DNMT1 overexpression (LVDNMT1) and HSP90AA1 overexpression (LVHSP90AA1) were acquired from Shanghai GeneChem Co. Ltd. Primary LCs were seeded into 6‐well plates at approximately 50% confluence. After cells adhered, 1 mL of medium and a final concentration of 5 μg/mL of polybrene were added. Subsequently, cells were incubated with shDNMT1 lentivirus (MOI = 10; 3.37 × 10^8^ TU/mL), LVFTO lentivirus (MOI = 80; 4 × 10^8^ TU/mL), shFTO lentivirus (MOI = 120; 6 × 10^8^ TU/mL), LVDNMT1 lentivirus (MOI = 50; 5.4 × 10^8^ TU/mL), or LVHSP90AA1 lentivirus (MOI = 50; 3.3 × 10^9^ TU/mL) along with corresponding negative controls. After 24 h, the medium was replaced, and cells were cultured for another 3 days.

### 2.11 Immunohistochemical Staining

Testicular tissues from three randomly selected rats per group were fixed, embedded in paraffin, and serially sectioned at a thickness of 4 μm along the transverse midline of each testis. Sections were dewaxed, rehydrated through a graded ethanol series, and subjected to antigen retrieval in citrate buffer (pH 6.0). Endogenous peroxidase activity was blocked with 3% H_2_O_2_. Sections were then incubated with primary antibodies (see Table 2) overnight at 4°C. The following day, detection was performed using the rabbit anti‐two‐step kit (PV‐6001; Zhongshan Golden Bridge) and DAB chromogen solution (ZLI‐9018; Zhongshan Golden Bridge). Sections from comparable regions of each rat testis were examined. Five random fields of view were photographed under a microscope and analyzed using ImageJ software.

**TABLE 2:** The details of antibodies used in the experiments.

Antibody	Source	Dilution
*For immunohistochemical staining*		
DNMT1	ABclonal, Cat. No. A22455, clone ARC51345	1:100
FTO	Proteintech, Cat. No. 27226‐1‐AP	1:100
HSP90AA1	Proteintech, Cat. No. 13171‐1‐AP	1:100
Caspase 3	Abways, Cat. No. CY5384	1:100
*For Western blot analysis*		
DNMT1	ABclonal, Cat. No. A22455, clone ARC51345	1:1000
FTO	Proteintech, Cat. No. 27226‐1‐AP	1:5000
HSP90AA1	Proteintech, Cat. No. 13171‐1‐AP	1:5000
StAR	Proteintech, Cat. No. 12225‐1‐AP	1:5000
CYP11A1	Solarbio, Cat. No. K002941P	1:1000
CYP17A1	Solarbio, Cat. No. K022567RR	1:1000
p16	Wanleibio, Cat. No. WL01418	1:1000
AKT	Wanleibio, Cat. No. WL0003b	1:500
p‐AKT	Wanleibio, Cat. No. WLP001a	1:500
BCL‐2	Wanleibio, Cat. No. WL01556	1:500
BAX	Wanleibio, Cat. No. BCL2L4	1:500
β‐Actin	Proteintech, Cat. No. 20536‐1‐AP	1:5000
Goat anti‐rabbit IgG (HRP)	Abways, Cat. No. AB0101	1:5000

### 2.12 TUNEL Staining

TUNEL staining was performed using the One‐step TUNEL In Situ Apoptosis Kit (Green, FITC; E‐CK‐A320; Elabscience) according to the manufacturer's instructions. Briefly, cells cultured on coverslips were fixed with 4% paraformaldehyde and permeabilized with 0.1% Triton X‐100. For paraffin‐embedded testicular sections, dewaxing, rehydration, and proteinase K permeabilization were performed prior to staining. After equilibration, samples were incubated with the TUNEL reaction mixture for labeling. Nuclei were counterstained with DAPI, and sections were mounted for visualization. Three sections per group were examined. Five random fields per section were captured under a fluorescence microscope, and the percentage of TUNEL‐positive cells was quantified using ImageJ software.

### 2.13 Western Blot

High‐efficiency tissue/cell lysate (R0010; Solarbio) was used to extract proteins. Protein concentration was measured using the BCA method (P0012S; Beyotime). Following SDS‐PAGE electrophoresis, proteins were transferred to PVDF membranes at 300 mA for 2 h. Membranes were blocked with 5% skim milk at room temperature for 1 h, followed by incubation with primary antibodies (see Table 2) overnight at 4°C. After washing, membranes were incubated with HRP‐conjugated goat anti‐rabbit IgG secondary antibody (AB0101; ABWAYS) at a dilution of 1:10,000 for 1.5 h at room temperature. Protein bands were visualized using an enhanced chemiluminescence (ECL) substrate (BL520A; Biosharp) and imaged with a chemiluminescence imaging system. Band intensities were quantified using ImageJ software, and relative protein expression was calculated as the ratio of the target protein band intensity to that of the reference protein (β‐actin).

### 2.14 Statistical Analysis

All data were analyzed using GraphPad Prism 5.0 statistical software. Data are presented as mean ± SEM from at least three independent biological replicates. All data underwent normality testing and met the criteria for normal distribution. Comparisons between two groups were analyzed using unpaired two‐tailed Student's *t*‐test. For multiple‐group comparisons, one‐way analysis of variance (ANOVA) was performed, followed by Dunnett's post hoc test for comparisons versus a single control group or Tukey's post hoc test for pairwise comparisons among all groups. Welch's correction was applied when homogeneity of variance was not satisfied. Statistically significant differences were defined as *p* < 0.05 (*p < 0.05, **p < 0.01, and ***p < 0.001). ns indicated no statistically significant difference.

## 3 Results

### 3.1 Expression Changes of DNMT1 and Testosterone in the Testes of Aging Rats and In Vitro Senescent Leydig Cells

DNA methylation is a pivotal epigenetic modification governing mammalian spermatogenesis and testicular homeostasis (Chen and Zhang 2020; Siebert‐Kuss et al. 2024). To dissect the association between DNA methyltransferases (DNMTs) activity, testosterone levels, and age‐related testicular dysfunction, we longitudinally profiled DNMTs activity and testosterone content in rat testicular tissues at different ages. The results revealed that both parameters exhibited pronounced age‐dependent trajectories, with a significant concurrent decline commencing at 12 months of age (Figure 1a,b). Given that testosterone production relies on the coordinated enzymatic activities of CYP11A1, CYP17A1, and StAR, we next examined their expression levels. The expression patterns of these synthases closely mirrored the temporal changes in testosterone content and DNMTs activity, all showing marked downregulation after 12 months of age (Figure 1c–e). Intriguingly, mining of existing human multi‐tissue transcriptomic datasets revealed that DNMT1, the principal member of the DNMT family, is preferentially and abundantly expressed in the testis (Dezso et al. 2008), positioning it as a prime candidate regulator of testicular function (Figure 1f). To validate this hypothesis in an aging context, we focused on DNMT1 expression dynamics in the rat testis. Complementary RT‐qPCR, Western blotting, and immunohistochemical staining consistently demonstrated a progressive age‐related decline in DNMT1 expression (Figure 1g–k).

**FIGURE 1:**
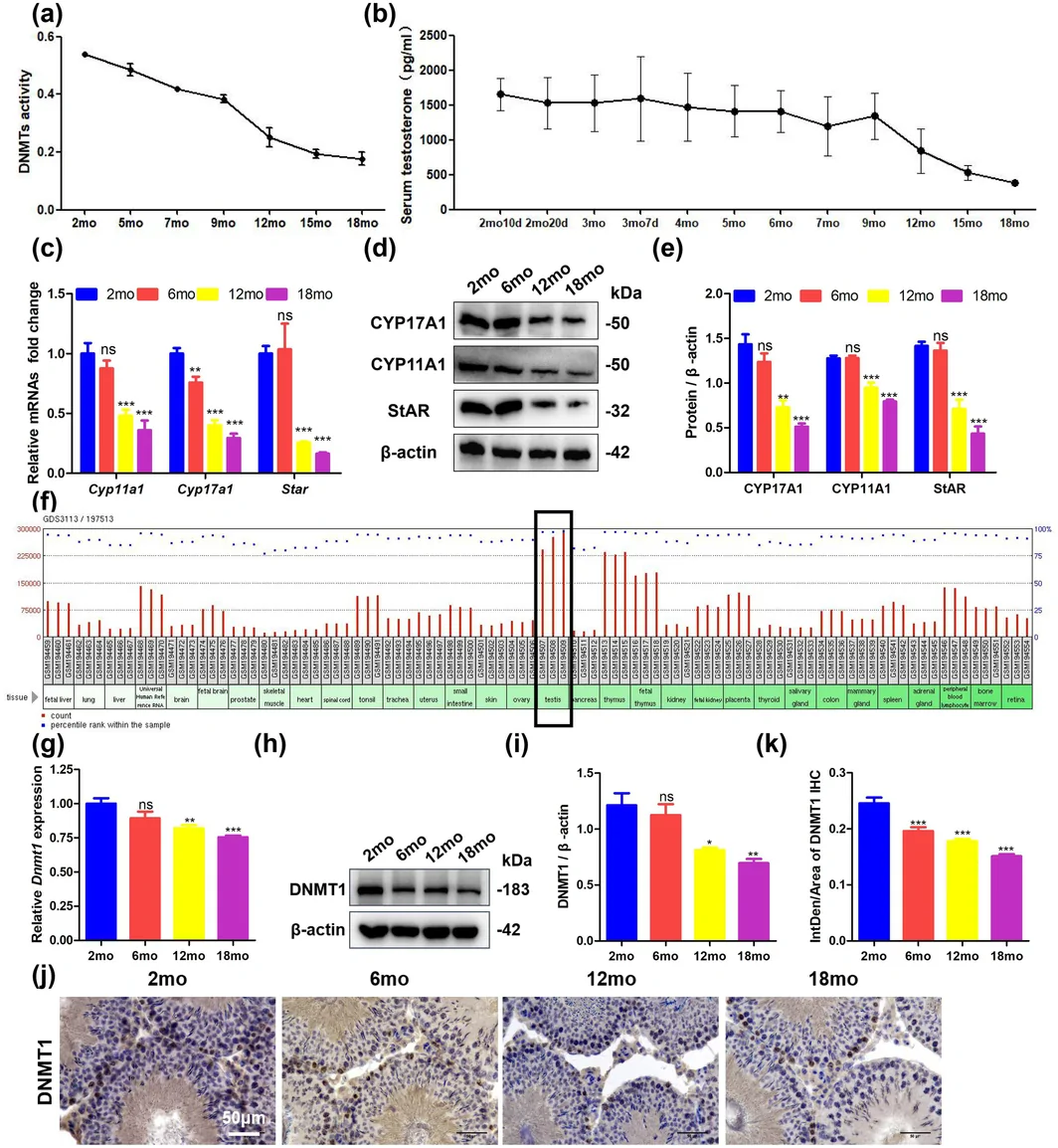
Age‐related decline of DNMT1 expression and testosterone levels in rat testes. (a) DNMT activity in rat testicular tissues measured by ELISA, showing an age‐dependent decreasing trend. (b) Serum testosterone levels in rats of different ages. (c) Relative mRNA expression of steroidogenic enzymes *Cyp11a1*, *Cyp17a1*, and *Star* in rat testes determined by RT‐qPCR. (d) Representative Western blot images of CYP11A1, CYP17A1, and StAR protein expression. (e) Densitometric quantification of Western blot bands in (d). (f) GEO data analysis of DNMT1 expression in different tissues. (g) Relative mRNA expression of *Dnmt1* in rat testes at different ages by RT‐qPCR. (h) Representative Western blot images of DNMT1 protein expression. (i) Densitometric quantification of DNMT1 Western blot bands. (j) Representative immunohistochemical staining of DNMT1 in rat testicular sections (scale bar = 50 μm). (k) Quantification of immunohistochemical staining intensity. *n* = 5 images from *n* = 3 independent experiments. Data are presented as mean ± SEM. *p < 0.05, **p < 0.01, ***p < 0.001 compared with 2‐month‐old rats (ANOVA and Dunnett's T3 test). *n* = 3 per group.

To further dissect the intrinsic relationship between cellular senescence and DNMT1 expression while excluding confounding influences from the in vivo microenvironment, we established an in vitro primary Leydig cell (LC) aging model. Primary LCs were isolated and purified from postnatal Day 15–20 Wistar rats and subjected to serial passaging to induce replicative senescence. Cell identity was first validated by 3β‐HSD specific staining, confirming the typical LC phenotype (Figure S1a). Senescence was assessed by SA‐β‐gal staining, which revealed that the blue‐positive cell rate in the aged group was significantly higher than in the control group, exceeding 70% positivity, indicating a robust senescent phenotype (Figure S1b,c). RT‐qPCR analysis demonstrated significant upregulation of the senescence marker p16 and senescence‐associated secretory phenotype (SASP) factors *Tnf*, *Il1b*, and *Il6* at the mRNA level in aged LCs compared to controls, with parallel increases in p16 protein expression confirmed by Western blotting (Figure S1d–f). Collectively, these data confirm the successful establishment of a stable in vitro LC aging model. In this model, we further observed that senescent LCs exhibited a marked reduction in testosterone production (Figure 1g), accompanied by significant downregulation of the key steroidogenic enzymes CYP11A1, CYP17A1, and StAR, as well as a pronounced decrease in DNMT1 expression (Figure S1h–j). These findings recapitulate the age‐related declines observed in vivo, supporting DNMT1 as a cell‐intrinsic correlate of LC functional aging.

Collectively, our findings from both in vivo and in vitro models demonstrate that aging is consistently associated with the concurrent decline of DNMT1 expression and testosterone biosynthesis, implicating DNMT1 as a candidate epigenetic node in the functional decay of the aging testis.

### 3.2 DNMT1 Positively Regulates Testosterone Synthesis and Cellular Senescence in Leydig Cells

To functionally interrogate the role of DNMT1 in LC testosterone synthesis, we employed lentivirus‐mediated knockdown and overexpression of DNMT1 in primary rat LCs. The efficacy of DNMT1 knockdown was first validated by Western blotting, which confirmed a significant reduction in DNMT1 protein levels in shDNMT1‐transduced cells compared to controls (Figure 2a,b). Functionally, DNMT1 knockdown led to a marked decrease in testosterone secretion into the culture supernatant, as measured by ELISA (Figure 2c). At the molecular level, knockdown of DNMT1 significantly suppressed the protein expression of key steroidogenic enzymes—CYP11A1, CYP17A1, and StAR—all of which are rate‐limiting components of the testosterone biosynthesis pathway (Figure 2d,e). Conversely, lentiviral‐mediated overexpression of DNMT1 produced the reciprocal phenotype, resulting in significantly elevated testosterone levels and upregulated expression of CYP11A1, CYP17A1, and StAR (Figure 2f–j). Collectively, these results confirm that DNMT1 acts as a positive regulator of LC testosterone synthesis in LCs.

**FIGURE 2:**
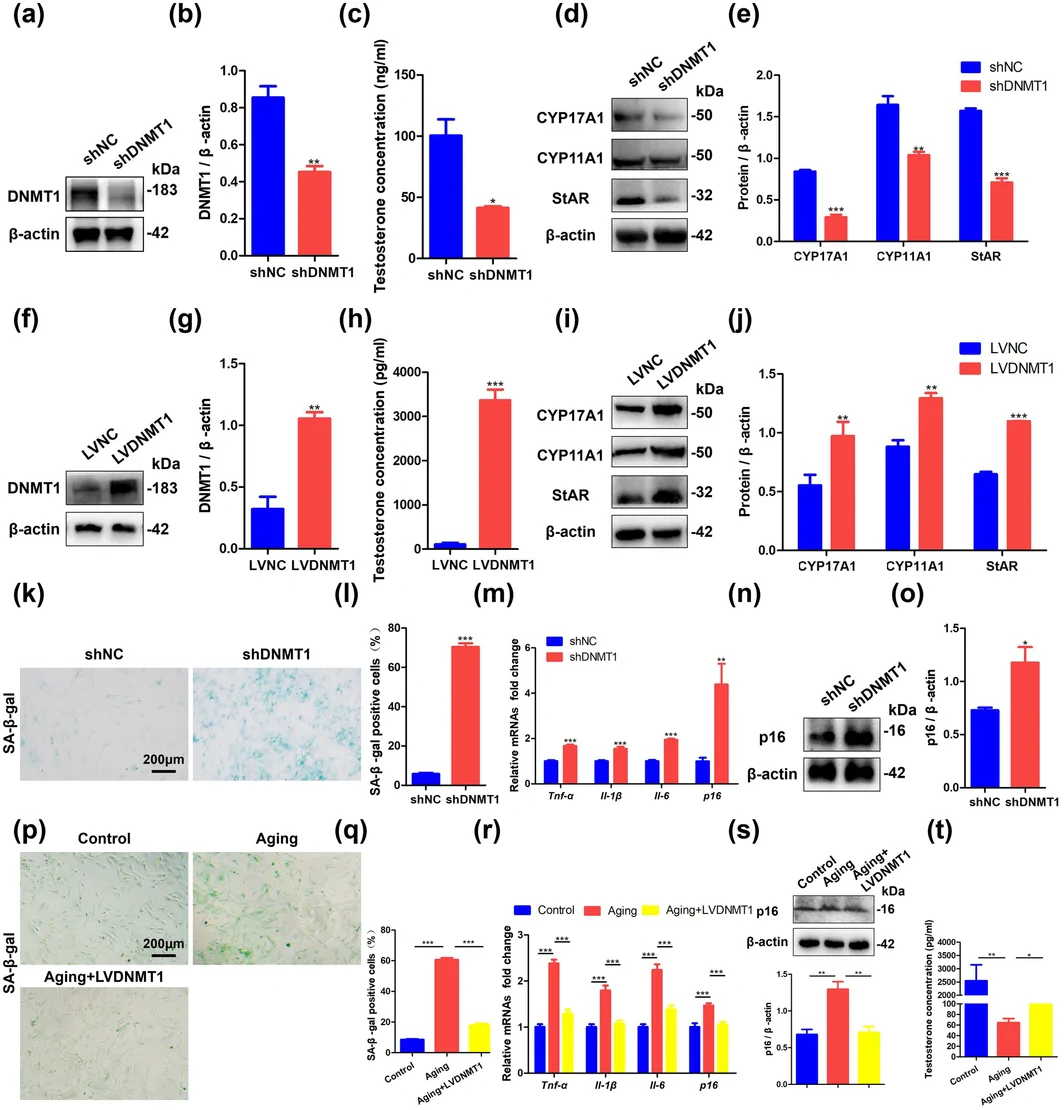
DNMT1 regulates testosterone synthesis and cellular senescence in Leydig cells. (a) Representative Western blot images of DNMT1 protein expression in LCs transduced with shNC or shDNMT1 (b) Western blot densitometric analysis of DNMT1. (c) Testosterone levels in the culture supernatant of LCs following DNMT1 knockdown, measured by ELISA. (d) Representative Western blot images of CYP11A1, CYP17A1, and StAR protein expression after DNMT1 knockdown. (e) Western blot densitometric analysis of CYP11A1, CYP17A1, and StAR. (f) Representative Western blot images of DNMT1 protein expression in LCs transduced with LV‐NC or LV‐DNMT1. (g) Densitometric quantification of DNMT1 protein levels. (h) Testosterone levels in the culture supernatant following DNMT1 overexpression. (i) Representative Western blot images of CYP11A1, CYP17A1, and StAR protein expression after DNMT1 overexpression. (j) Densitometric quantification of protein bands shown in (i). (k) Representative β‐galactosidase staining image (scale bar = 200 μm). (l) Percentage of SA‐β‐gal‐positive cells, calculated from 5 random fields per group across 3 independent experiments. (m) Relative mRNA expression of SASP factors (*Tnf*, *Il1b*, *Il6*) and the senescence marker *p16* in LCs, determined by RT‐qPCR. (n) Representative Western blot images of p16 protein expression after DNMT1 knockdown. (o) Densitometric quantification of p16 protein levels. (p) Representative images of SA‐β‐gal staining in senescent LCs with or without DNMT1 overexpression (scale bar = 200 μm). (q) Quantification of SA‐β‐gal‐positive cells per field. (r) Relative mRNA expression of *Tnf*, *Il1b*, *Il6*, and *p16* in senescent LCs following DNMT1 overexpression. (s) Representative Western blot images of p16 protein expression in senescent LCs with DNMT1 overexpression. (t) Testosterone levels in the culture supernatant of senescent LCs after DNMT1 overexpression. Data are presented as mean ± SEM. *p < 0.05, **p < 0.01, ***p < 0.001 compared with shNC or LV‐NC group (Student's *t‐*test for b, c, e, g, h, j, l, m, o); *p < 0.05, **p < 0.01, ***p < 0.001 compared with aging group (ANOVA with Dunnett's T3 test for q, r, s, t). *n* = 3 per group.

In addition, DNMT1 knockdown significantly increased the proportion of SA‐β‐gal‐positive cells (Figure 2k,l) and upregulated the expression of the senescence marker p16 as well as the SASP factors *Tnf*, *Il1b*, and *Il6* (Figure 2m–o), indicating that DNMT1 deficiency effectively drives LC senescence. Conversely, DNMT1 overexpression in senescent LCs reversed this phenotype, as evidenced by a reduced SA‐β‐gal‐positive cell rate, decreased expression of *Tnf*, *Il1b*, and *Il6*, and p16 (Figure 2p–s), and restored testosterone secretion (Figure 2t). Collectively, these results demonstrate that DNMT1 not only positively regulates testosterone biosynthesis but also suppresses the cellular senescence program in LCs, positioning DNMT1 as a potential epigenetic safeguard against functional decline in the aging testis.

### 3.3 DNMT1 Represses FTO Expression via Promoter Methylation to Regulate Testosterone Synthesis

To elucidate the molecular mechanism by which DNMT1 regulates testosterone synthesis, we performed RNA sequencing (RNA‐seq) on DNMT1‐knockdown LCs to identify differentially expressed genes. Among the altered transcripts, the mRNA encoding the m^6^A demethylase FTO (fat mass and obesity‐associated protein) was significantly upregulated in shDNMT1 LCs compared with shNC controls (Figure 3a). Given that FTO is a well‐established m^6^A demethylase involved in mRNA splicing, translation, and stability, we next investigated whether DNMT1 regulates FTO expression at the epigenetic level. Methylation‐specific PCR (MSP) revealed that the methylation level of the *Fto* promoter region was significantly reduced following DNMT1 knockdown (Figure 3b,c). Chromatin immunoprecipitation coupled with quantitative PCR (ChIP‐qPCR) further confirmed that DNMT1 specifically binds to the CpG island within the *Fto* promoter, with an enrichment fold of approximately 398‐fold over the IgG control (Figure 3d). Complementary RT‐qPCR and Western blot analyses consistently demonstrated that DNMT1 knockdown markedly upregulated FTO expression at both mRNA and protein levels, whereas DNMT1 overexpression significantly suppressed FTO expression (Figure 3e–j). Collectively, these data indicate that DNMT1 negatively regulates FTO expression by promoting methylation of the *Fto* promoter.

**FIGURE 3:**
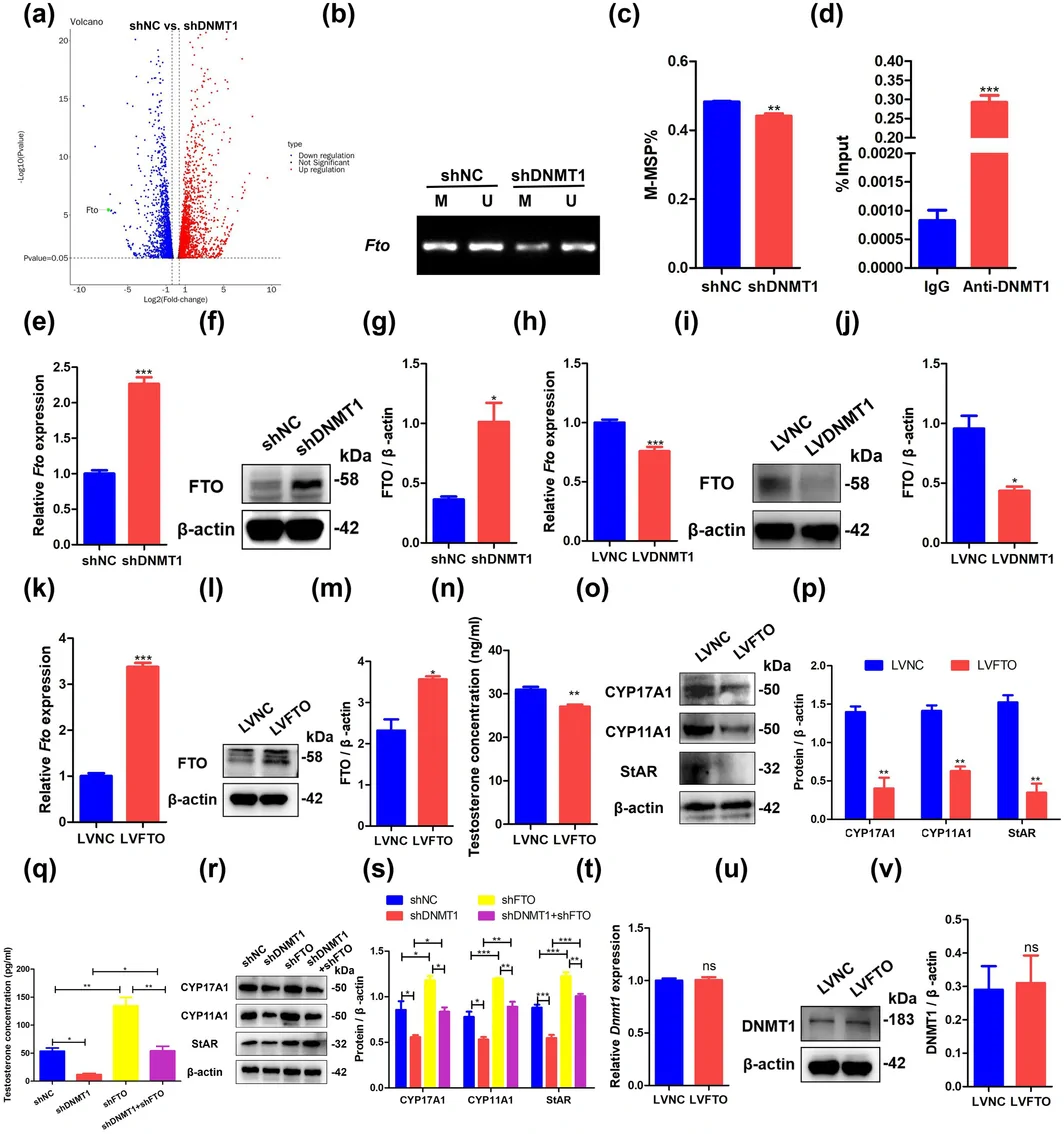
DNMT1 represses FTO expression via promoter methylation without reciprocal regulation by FTO. (a) RNA‐seq analysis showing differential expression of FTO (highlighted) in shDNMT1 versus shNC LCs. (b) MSP analysis of *Fto* promoter methylation status following DNMT1 knockdown; M: methylated; U: unmethylated. (c) MSP statistical results. (d) ChIP‐qPCR analysis of DNMT1 occupancy at the Fto promoter CpG island, presented as enrichment relative to IgG control. (e, f) RT‐qPCR (e) and Western Blot (f) results show upregulation of FTO expression after DNMT1 knockdown. (g) Western blot densitometric analysis of DNMT1. (h, i) RT‐qPCR (h) and Western Blot (i) analysis showing downregulation of FTO expression following DNMT1 overexpression. (j) Densitometric quantification of FTO protein levels. (k, l) RT‐qPCR (k) and Western blot (l) validation of FTO overexpression efficiency. (m) Densitometric quantification of FTO protein levels. (n) Testosterone content decreases after FTO overexpression. (o) Representative Western blot images of CYP11A1, CYP17A1, and StAR protein expression after FTO overexpression. (p) Densitometric quantification of protein bands shown in (o). (q) Testosterone levels in shNC, shDNMT1, shFTO, and shDNMT1 + shFTO groups. (r) Representative Western blot images of CYP11A1, CYP17A1, and StAR expression in single and dual knockdown groups. (s) Densitometric quantification of protein bands shown in (r). (t) RT‐qPCR shows no change in Dnmt1 mRNA levels. (u) Western Blot analysis showed no change in DNMT1 protein levels. (v) Western blot densitometric analysis of DNMT1. Data are presented as mean ± SEM. *p < 0.05, **p < 0.01, ***p < 0.001 compared with shNC group or LVNC group (Student's *t*‐test for c–e, g, h, j, k, m, n, p, t, v). *p < 0.05, **p < 0.01, ***p < 0.001 compared with shNC or shFTO or shDNMT1 group (ANOVA and Tukey test for q, s). *n* = 3 per group. ns, not significant.

To determine the functional relationship between FTO and testosterone synthesis, we established an FTO‐overexpressing LC model, which was validated by RT‐qPCR and Western blotting (Figure 3k–m). FTO overexpression significantly reduced testosterone levels in the culture supernatant and downregulated the expression of CYP11A1, CYP17A1, and StAR (Figure 3n–p), phenocopying the effect of DNMT1 knockdown. To further test whether DNMT1 modulates testosterone synthesis in an FTO‐dependent manner, we performed dual knockdown of DNMT1 and FTO in LCs. Compared with DNMT1 knockdown alone, the concurrent knockdown of DNMT1 and FTO significantly restored testosterone production and rescued the expression of steroidogenic enzymes. Conversely, relative to FTO knockdown alone, the dual knockdown resulted in a marked reduction in testosterone synthesis and key enzyme expression (Figure 3q–s). Of note, FTO overexpression did not significantly affect DNMT1 expression (Figure 3t–v), suggesting that FTO does not exert feedback regulation on DNMT1. Taken together, these findings establish that DNMT1 positively regulates testosterone synthesis by promoting FTO promoter methylation to repress FTO expression, and that this regulatory axis is unidirectional, with no reciprocal regulation of DNMT1 by FTO.

### 3.4 FTO Modulates HSP90AA1 Expression via m^6^A Modification, With Indirect Regulation by DNMT1 Through FTO

As a key m^6^A demethylase, FTO can modulate the m^6^A modification status of target gene transcripts, thereby influencing gene expression. To identify potential downstream targets of FTO in LCs, we first performed in silico analysis using the catRAPID protein‐RNA interaction prediction database to assess the binding propensity of rat FTO protein with candidate gene mRNAs. Among the candidates, Hsp90aa1 mRNA exhibited the highest interaction propensity score with FTO. Moreover, two RNA‐binding domains were identified within the FTO protein sequence, with detailed binding sites depicted in Figure 4a. Consistent with our previous m^6^A microarray screening, the m^6^A level of *Hsp90aa1* transcripts was significantly reduced in aging testicular tissues (Figure 4b). To experimentally validate the direct interaction between FTO protein and *Hsp90aa1* transcripts, we performed RNA immunoprecipitation coupled with quantitative PCR (RIP‐qPCR) using an FTO‐specific antibody versus IgG control. The results confirmed that *Hsp90aa1* mRNA is a direct binding substrate of FTO (Figure 4c).

**FIGURE 4:**
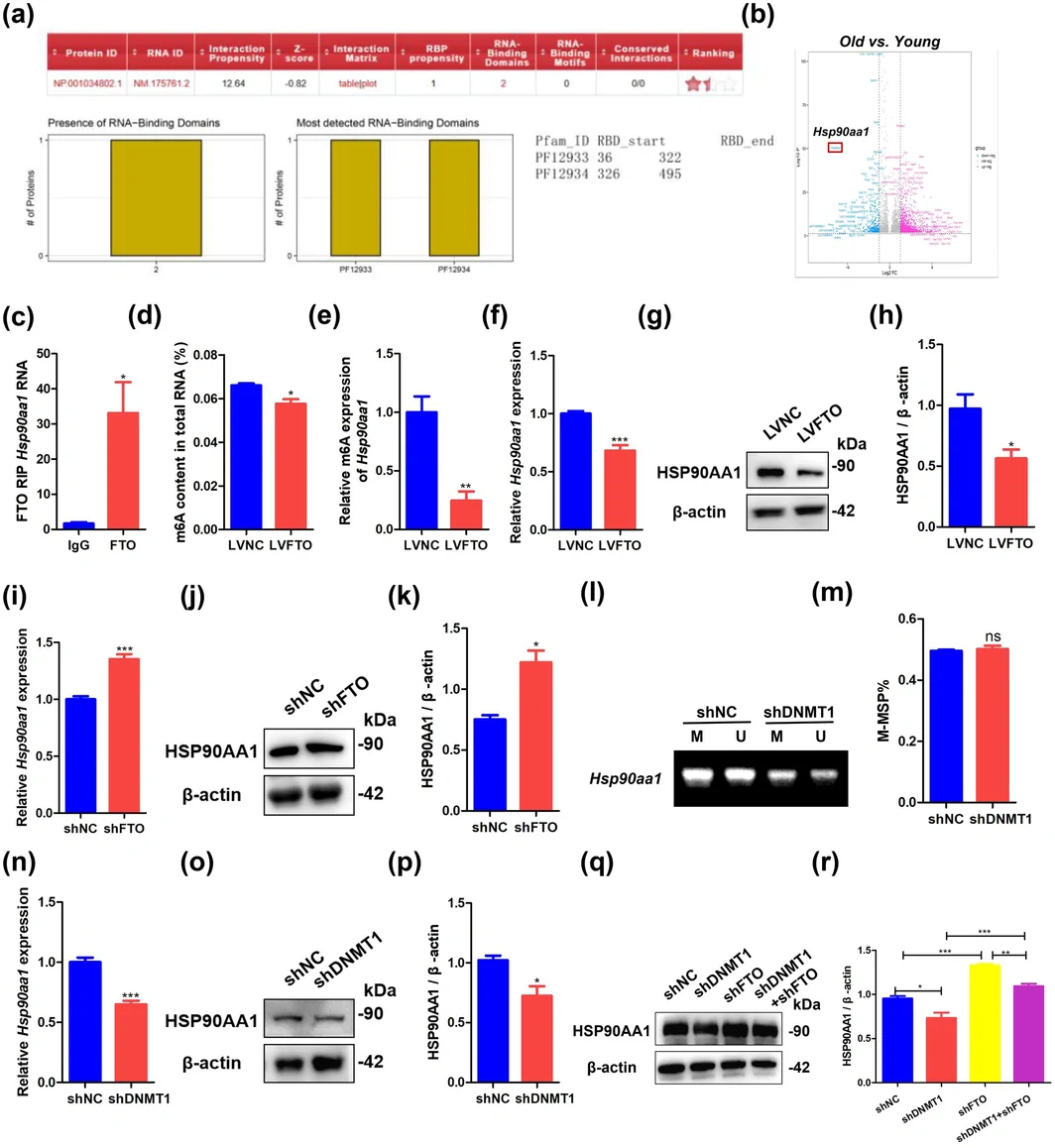
FTO modulates HSP90AA1 expression via m^6^A modification, with indirect regulation by DNMT1 through FTO. (a) catRAPID in silico prediction showing potential binding sites between FTO and *Hsp90aa1* mRNA, with the interaction propensity score indicated. (b) m^6^A microarray analysis revealing reduced m^6^A levels of *Hsp90aa1* transcripts in aging versus young testicular tissues. (c) RIP‐qPCR results confirm that *Hsp90aa1* mRNA is a direct binding substrate of FTO, presented as enrichment relative to IgG control. (d, e) Overexpression of FTO in LCs decreased total m^6^A levels (d) and m^6^A levels on the *Hsp90aa1* gene (e). (f, g) RT‐qPCR (f) and Western Blot (g) results showed downregulated HSP90AA1 expression in the LVFTO group. (h) Western blot densitometric analysis of HSP90AA1. (i, j) RT‐qPCR (i) and Western Blot (j) results show that HSP90AA1 expression is upregulated in the shFTO group. (k) Western blot densitometric analysis of HSP90AA1. (l) MSP analysis of *Hsp90aa1* promoter DNA methylation status after DNMT1 knockdown; M: methylated; U: unmethylated. (m) MSP statistical results. (n, o) RT‐qPCR (n) and Western Blot (o) results show downregulated HSP90AA1 expression in the shDNMT1 group. (p) Western blot densitometric analysis of HSP90AA1. (q) Representative Western blot images of HSP90AA1 protein expression in shNC, shDNMT1, shFTO, and shDNMT1 + shFTO groups. (r) Densitometric quantification of HSP90AA1 protein bands shown in (q). Data are presented as mean ± SEM. *p < 0.05, **p < 0.01, ***p < 0.001 compared with LVNC group or shNC group (Student's *t*‐test for c–f, h, i, k, m, n, p). *p < 0.05, **p < 0.01, ***p < 0.001 compared with shNC or shFTO or shDNMT1 group (ANOVA and Tukey test for r). *n* = 3 per group. ns, not significant.

To determine the regulatory effect of FTO on HSP90AA1 expression, we transduced primary LCs with lentiviral vectors for FTO overexpression. FTO overexpression significantly decreased both global cellular m^6^A levels and the m^6^A modification level of *Hsp90aa1* transcripts (Figure 4d,e), accompanied by a concurrent downregulation of *Hsp90aa1* mRNA and HSP90AA1 protein expression (Figure 4f–h). Conversely, FTO knockdown markedly upregulated *Hsp90aa1* mRNA and HSP90AA1 protein levels in LCs (Figure 4i–k), confirming that FTO negatively regulates HSP90AA1 expression.

We next investigated whether DNMT1 directly regulates HSP90AA1 through promoter DNA methylation. DNMT1 knockdown did not significantly alter the DNA methylation level of the *Hsp90aa1* promoter (Figure 4l,m), yet both *Hsp90aa1* mRNA and HSP90AA1 protein expression were markedly reduced in DNMT1‐deficient LCs (Figure 4n–p). Notably, simultaneous FTO knockdown in DNMT1‐knockdown LCs effectively rescued the suppressive effect of DNMT1 deficiency on HSP90AA1 expression (Figure 4q,r), indicating that the downregulation of HSP90AA1 upon DNMT1 loss is mediated through FTO rather than direct promoter methylation. Collectively, these findings demonstrate that FTO modulates HSP90AA1 expression by altering the m^6^A modification status of its transcripts, and that DNMT1 indirectly regulates HSP90AA1 expression via the FTO axis, independent of DNA methylation at the *Hsp90aa1* locus.

### 3.5 The DNMT1–FTO Axis Inhibits Leydig Cell Apoptosis and Promotes Testosterone Synthesis via HSP90AA1‐Mediated AKT Signaling

HSP90AA1 functions as a molecular chaperone for AKT, promoting its stability and activation, thereby modulating diverse biological processes (Zhang et al. 2023). Given that AKT signaling is a well‐established anti‐apoptotic pathway also implicated in testosterone secretion (Li, Yao, et al. 2023; Sun et al. 2022), we hypothesized that HSP90AA1 suppresses LC apoptosis through AKT activation, thereby promoting testosterone synthesis. To test this hypothesis, we established a lentivirus‐mediated HSP90AA1 overexpression model in primary LCs, which was validated by Western blotting (Figure 5a,b). HSP90AA1 overexpression significantly enhanced testosterone production and upregulated the expression of key steroidogenic enzymes, including CYP17A1, CYP11A1, and StAR (Figure 5c–e). Mechanistically, HSP90AA1 overexpression did not alter total AKT protein levels but markedly increased phosphorylated AKT (p‐AKT) and the anti‐apoptotic protein BCL‐2, whereas reducing the pro‐apoptotic protein BAX (Figure 5f,g). Notably, HSP90AA1 overexpression exerted no significant effect on FTO or DNMT1 expression (Figure 5h,i), indicating that HSP90AA1 acts downstream of the DNMT1–FTO axis. Collectively, these results demonstrate that HSP90AA1 activates AKT signaling, attenuates LC apoptosis, and ultimately facilitates testosterone synthesis.

**FIGURE 5:**
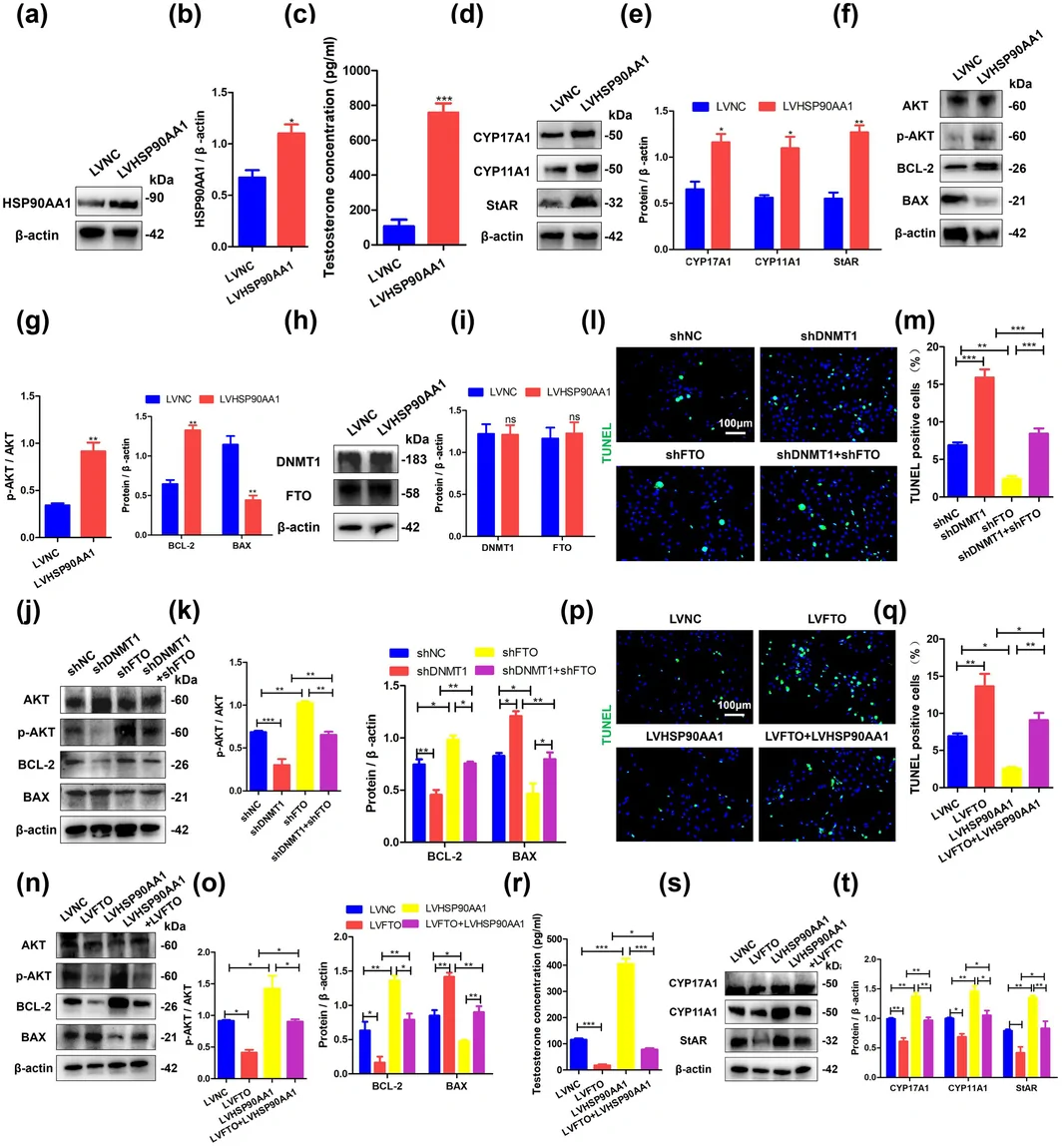
The DNMT1–FTO axis inhibits Leydig cell apoptosis and promotes testosterone synthesis via HSP90AA1‐mediated AKT signaling. (a) Representative Western blot images validating HSP90AA1 overexpression efficiency in LCs transduced with LV‐NC or LV‐HSP90AA1. (b) Densitometric quantification of HSP90AA1 protein levels. (c) Testosterone levels in the culture supernatant measured by ELISA. (d) Representative Western blot images showing upregulation of CYP11A1, CYP17A1, StAR, and DNMT1 protein expression following HSP90AA1 overexpression. (e) Densitometric quantification of CYP11A1, CYP17A1, and StAR protein levels. (f) Representative Western blot images showing increased p‐AKT and BCL‐2, with decreased BAX expression, in LV‐HSP90AA1 LCs. (g) Densitometric quantification of p‐AKT, BCL‐2, and BAX protein levels. (h) Representative Western blot images showing that HSP90AA1 overexpression does not affect DNMT1 or FTO expression. (i) Densitometric quantification of DNMT1 and FTO protein levels. Data in (a–i) are presented as mean ± SEM. *p < 0.05, **p < 0.01, ***p < 0.001 compared with LV‐NC (Student's *t‐*test); *n* = 3 per group. (j) Representative Western blot images of p‐AKT, BCL‐2, and BAX expression in shNC, shDNMT1, shFTO, and shDNMT1 + shFTO groups. (k) Densitometric quantification of protein bands shown in (j). (l) Representative images of TUNEL staining in the above four groups (scale bar = 50 μm). (m) Quantification of TUNEL‐positive cells, expressed as percentage of total cells. (n) Representative Western blot images of p‐AKT, BCL‐2, and BAX expression in LV‐NC, LV‐FTO, LV‐HSP90AA1, and LV‐FTO + LV‐HSP90AA1 groups. (o) Densitometric quantification of protein bands shown in (n). (p) Representative TUNEL staining images in the above four groups (scale bar = 50 μm). (q) Quantification of TUNEL‐positive cell percentage. (r) Testosterone levels in the culture supernatant measured by ELISA. (s) Representative Western blot images of CYP11A1, CYP17A1, and StAR expression in LV‐NC, LV‐FTO, LV‐HSP90AA1, and LV‐FTO + LV‐HSP90AA1 groups. (t) Densitometric quantification of protein bands shown in (s). Data in (j–t) are presented as mean ± SEM. *p < 0.05, **p < 0.01, ***p < 0.001 compared with indicated groups (ANOVA and Tukey test). *n* = 3 per group.

To further validate the functional role of HSP90AA1, primary LCs were treated with the HSP90AA1 inhibitor 17‐AAG. Combined CCK‐8 and Western blot analyses established the optimal intervention conditions as 0.5 μM for 48 h (Figure S2a–c). Pharmacological inhibition of HSP90AA1 significantly reduced testosterone secretion and decreased the expression of StAR, CYP11A1, and CYP17A1 (Figure S2d–f). Pathway analysis revealed that p‐AKT and BCL‐2 levels were markedly downregulated, whereas BAX expression was significantly upregulated (Figure S2g,h). Consistent with the overexpression data, 17‐AAG‐mediated HSP90AA1 inhibition did not affect FTO or DNMT1 expression (Figure S2i,j).

To unequivocally establish the hierarchical relationship of the DNMT1–FTO–HSP90AA1 axis, we performed a series of rescue experiments. Compared with DNMT1 knockdown alone, dual knockdown of DNMT1 and FTO restored p‐AKT and BCL‐2 expression while reducing BAX expression. Conversely, relative to FTO knockdown alone, the dual knockdown reduced p‐AKT and BCL‐2 levels while elevating BAX expression (Figure 5j,k). TUNEL staining corroborated these molecular changes: the number of apoptotic positive cells in the dual‐knockdown group was lower than that in the DNMT1 knockdown group but higher than that in the FTO knockdown group (Figure 5l,m). The corresponding changes in testosterone levels and steroidogenic enzyme (StAR, CYP11A1, and CYP17A1) expression mirrored the above molecular trends, as described in Figure 3q–s.

In a complementary approach, we simultaneously overexpressed FTO and HSP90AA1 in LCs. The co‐overexpression group exhibited lower p‐AKT and BCL‐2 levels than the HSP90AA1 single‐overexpression group, yet higher levels than the FTO single‐overexpression group; BAX expression displayed the reciprocal pattern (Figure 5n,o). TUNEL staining further confirmed that the apoptotic rate in the co‐overexpression group was higher than in the HSP90AA1‐overexpressing group and lower than in the FTO‐overexpressing group (Figure 5p,q). Correspondingly, testosterone levels and CYP17A1, CYP11A1, and StAR expression in the co‐overexpression group were lower than in the HSP90AA1‐overexpressing group but higher than in the FTO‐overexpressing group (Figure 5r–t).

Finally, FTO‐knockdown LCs were treated with 17‐AAG to inhibit HSP90AA1. Compared with 17‐AAG treatment alone, the combined intervention significantly restored p‐AKT and BCL‐2 expression and reduced BAX levels (Figure S2k,l). Collectively, these multi‐layered rescue experiments establish that the DNMT1–FTO axis modulates testosterone synthesis in LCs by regulating HSP90AA1‐mediated AKT anti‐apoptotic signaling.

### 3.6 Age‐Related Alterations of the FTO–HSP90AA1–AKT Axis in Aging Rat Testes and Senescent Leydig Cells

Having established that the DNMT1–FTO axis suppresses LC apoptosis and promotes testosterone synthesis via HSP90AA1‐mediated AKT signaling (Sections 3.1, 3.5), we next sought to determine whether this regulatory axis is aberrantly altered during testicular aging. To this end, we examined the expression and activity of key components of the FTO–HSP90AA1–AKT pathway in both aging rat testes and senescent LCs in vitro.

In vivo, MSP analysis revealed that the methylated/unmethylated band ratio at the Fto promoter was significantly reduced in testicular tissues of 18‐month‐old rats compared with 2‐month‐old young controls (Figure 6a,b), indicating age‐associated *Fto* promoter hypomethylation. RIP‐qPCR demonstrated that FTO binding to *Hsp90aa1* mRNA was markedly enhanced in aged testes (Figure 6c). MeRIP‐qPCR further confirmed that the m^6^A modification level of *Hsp90aa1* transcripts was significantly decreased in 18‐month‐old rat testes (Figure 6d). Consistent with these findings, RT‐qPCR and Western blot analyses revealed that FTO expression was progressively upregulated with age, whereas HSP90AA1 expression was significantly downregulated during aging (Figure 6e–g). Immunohistochemical staining corroborated these results, showing elevated FTO, reduced HSP90AA1, and increased pro‐apoptotic Caspase3 expression in aged testicular sections (Figure 6h,i). TUNEL staining further verified a significant age‐dependent increase in apoptotic cells within testicular tissues (Figure 6j,k). Moreover, Western blot analysis demonstrated that p‐AKT and BCL‐2 protein levels were significantly decreased, whereas BAX was markedly upregulated in aging testes (Figure 6l,m).

**FIGURE 6:**
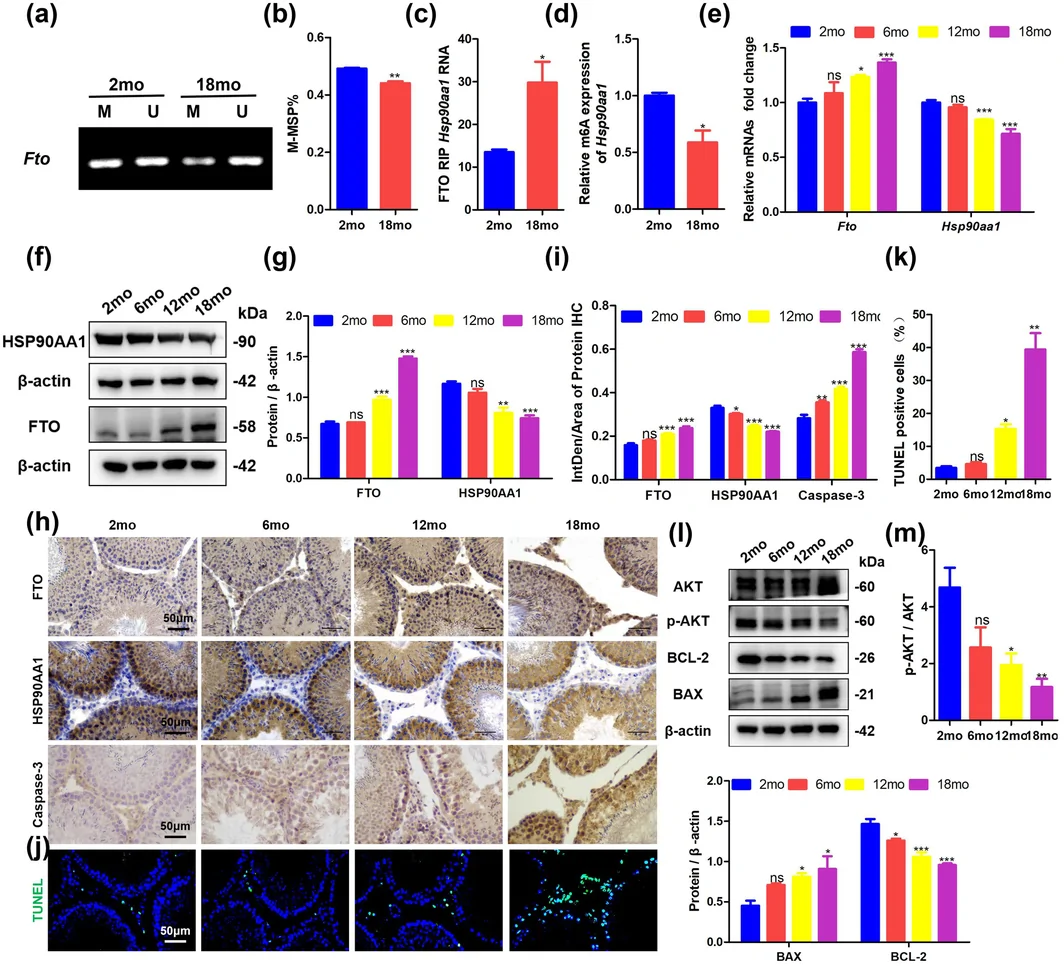
Age‐related alterations of the FTO–HSP90AA1–AKT axis in rat testes. (a) MSP analysis of *Fto* promoter methylation status in testicular tissues; M: methylated; U: unmethylated. (b) MSP statistical results. (c) RIP‐qPCR analysis showing FTO enrichment on *Hsp90aa1* mRNA in testicular tissues from 18‐month‐old versus 2‐month‐old rats. (d) MeRIP‐qPCR analysis of m^6^A modification levels of *Hsp90aa1* transcripts in testicular tissues of 2‐month‐old and 18‐month‐old rats. (e, f) RT‐qPCR (e) and Western blot (f) analysis revealing age‐dependent upregulation of FTO and downregulation of HSP90AA1 in rat testes. (g) Densitometric quantification of FTO and HSP90AA1 protein levels. (h) Representative immunohistochemical staining images of FTO, HSP90AA1, and Caspase‐3 in testicular sections (scale bar = 50 μm). (i) Quantification of immunohistochemical staining intensity. (j) Representative TUNEL staining images in testicular sections from rats at indicated ages (scale bar = 50 μm). (k) Quantification of TUNEL‐positive cells, expressed as percentage of total cells. (l) Representative Western blot images of p‐AKT, total AKT, BCL‐2, and BAX expression in testicular tissues from rats of different ages. (m) Densitometric quantification of p‐AKT, BCL‐2, and BAX protein levels (p‐AKT normalized to total AKT). Data are presented as mean ± SEM. *p < 0.05, **p < 0.01, ***p < 0.001 compared with 2 months. Student's *t*‐test for (b–d); ANOVA and Dunnett's T3 test for (e, j, i, k, m). *n* = 3 per group.

In vitro senescent LCs recapitulated the molecular alterations observed in vivo. Compared with control cells, senescent LCs exhibited reduced *Fto* promoter methylation (Figure S3a,b) and significantly elevated FTO mRNA and protein expression (Figure S3c–e). Both global cellular m^6^A levels and *Hsp90aa1*‐specific m^6^A modification were prominently decreased in senescent LCs (Figure S3f,g), accompanied by significant downregulation of *Hsp90aa1* mRNA and HSP90AA1 protein (Figure S3h–j). To further validate the functional relevance of this axis during physiological aging, primary LCs were isolated from 2‐month‐old (young) and 18‐month‐old (aged) rats. 3β‐HSD staining confirmed the purity of isolated LCs (Figure S4a), and SA‐β‐gal staining demonstrated a significantly higher proportion of senescent cells in the aged group (Figure S4b,c). Testosterone secretion was markedly reduced in LCs from aged rats (Figure S4d). Mechanistically, aged primary LCs displayed decreased *Fto* promoter methylation (Figure S4e,f) and reduced m^6^A modification of *Hsp90aa1* transcripts (Figure S4g). RT‐qPCR and Western blot analyses confirmed significantly lower DNMT1 and HSP90AA1 expression, alongside higher FTO expression, in the aged group (Figure S4h–j). Concomitantly, p‐AKT and BCL‐2 protein levels were decreased, whereas BAX expression was increased, in aged LCs (Figure S4k,l).

Collectively, both in vivo and in vitro data consistently demonstrate aberrant activation of the FTO–HSP90AA1–AKT axis during testicular aging. Mechanistically, age‐dependent hypomethylation of the *Fto* promoter leads to FTO upregulation, which in turn reduces the m^6^A modification level of *Hsp90aa1* transcripts and subsequently suppresses HSP90AA1 expression. These molecular events ultimately impair AKT anti‐apoptotic signaling, aggravate LC apoptosis, and culminate in diminished testosterone synthesis during testicular aging. Together with our mechanistic findings (Sections 3.2, 3.5), these observations establish a comprehensive model in which the DNMT1–FTO–HSP90AA1–AKT axis governs age‐related testicular functional decline.

### 3.7 In Vivo Knockdown of DNMT1 Validates the Regulatory Role of the DNMT1vFTO–HSP90AA1–AKT Axis in Testosterone Synthesis

To further substantiate the mechanistic findings obtained from in vitro experiments, we employed an adeno‐associated virus serotype 9 (AAV9)‐mediated gene delivery system to achieve in vivo DNMT1 knockdown specifically in testicular tissue via local intratesticular injection. To determine the optimal viral titer for efficient gene silencing, three graded doses of AAV9‐DNMT1 (8 × 10^9^, 4 × 10^10^, and 2 × 10^11^ vg) were administered, and testicular tissues were harvested 3 weeks post‐injection. No obvious morphological differences (Figure S5a) or changes in testicular index (Figure S5b) were observed between the AAV‐DNMT1 and AAV‐NC groups, indicating that DNMT1 knockdown did not grossly affect testicular architecture. RT‐PCR, Western blot, and immunohistochemistry results all showed the most significant reduction in DNMT1 expression in the high‐titer group (Figure S5c–g); this titer was therefore selected for subsequent in vivo interventions.

Following 3 weeks of AAV‐mediated DNMT1 knockdown, we assessed multiple functional and molecular readouts. Serum testosterone levels were significantly reduced in the AAV‐DNMT1 group compared with the AAV‐NC group (Figure 7a). Consistently, Western blot analysis confirmed that DNMT1 knockdown markedly downregulated the protein expression of the key steroidogenic enzymes CYP11A1, CYP17A1, and StAR (Figure 7b,c). To verify the in vivo operation of the regulatory axis, MSP analysis revealed that *Fto* promoter methylation was significantly decreased in the AAV‐DNMT1 group (Figure 7d,e). Combined RT‐qPCR, Western blot, and immunohistochemical analyses demonstrated that FTO expression was significantly upregulated, whereas HSP90AA1 expression was prominently downregulated, following in vivo DNMT1 knockdown (Figure 7f–j). Moreover, TUNEL staining revealed a marked increase in apoptotic cells within DNMT1‐deficient testicular tissues (Figure 7i,j). Concurrently, p‐AKT and BCL‐2 protein levels were significantly reduced, whereas BAX expression was increased, in the AAV‐DNMT1 group (Figure 7k,l). These in vivo observations are highly consistent with our in vitro findings, collectively demonstrating that the DNMT1–FTO axis modulates testosterone synthesis through HSP90AA1‐mediated AKT anti‐apoptotic signaling.

**FIGURE 7:**
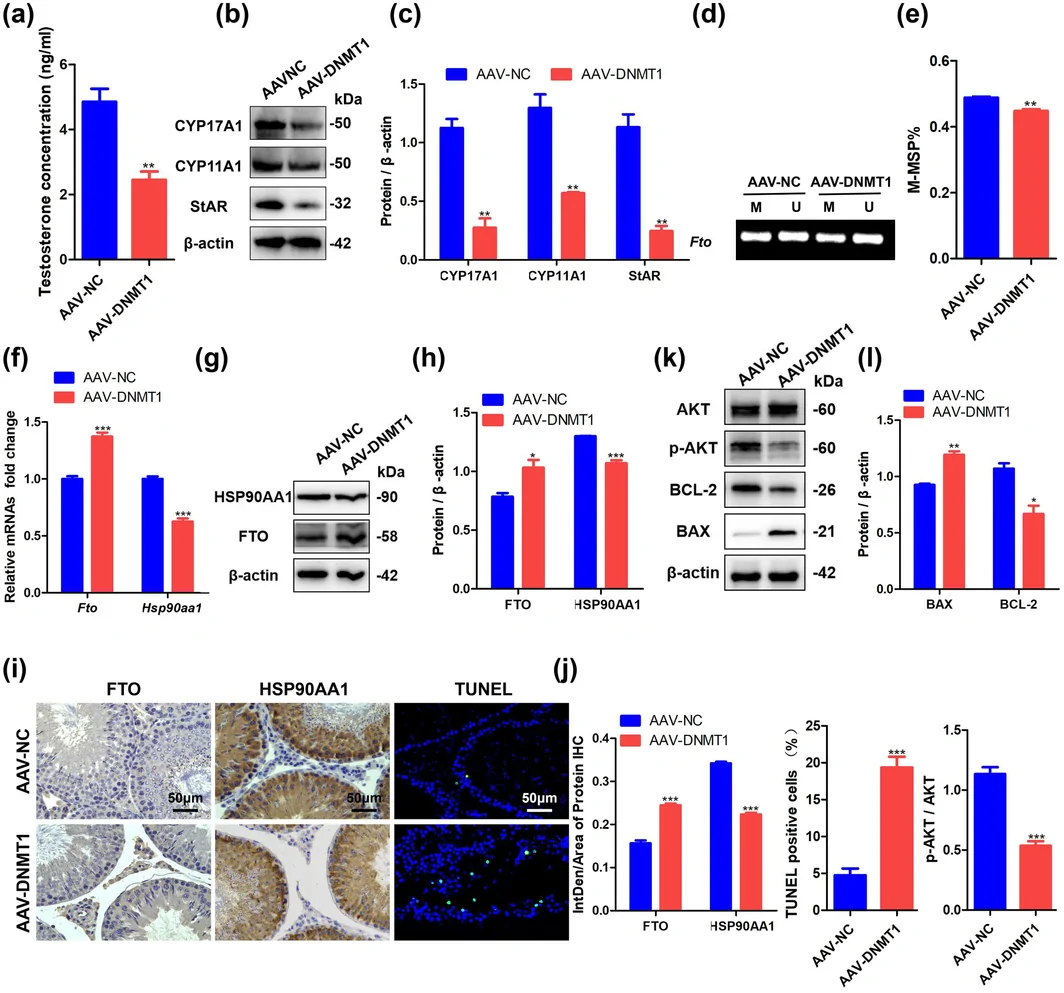
In vivo knockdown of DNMT1 validates the regulatory role of the DNMT1–FTO–HSP90AA1–AKT axis in testosterone synthesis. (a) Serum testosterone levels in AAV‐NC and AAV‐DNMT1 groups measured by ELISA. (b) Representative Western blot images showing downregulation of CYP11A1, CYP17A1, and StAR protein expression following in vivo DNMT1 knockdown. (c) Densitometric quantification of protein bands shown in (b). (d) MSP analysis of *Fto* promoter methylation status in testicular tissues from AAV‐NC and AAV‐DNMT1 groups; M: methylated; U: unmethylated. (e) MSP statistical results. (f–h) DNMT1 knockdown upregulates FTO and downregulates HSP90AA1. (i) Representative images of immunohistochemical staining (FTO and HSP90AA1) and TUNEL staining in testicular sections from AAV‐NC and AAV‐DNMT1 groups (scale bar = 50 μm). (j) Quantification of immunohistochemical staining intensity and TUNEL‐positive cell percentage. (k) Representative Western blot images of p‐AKT, total AKT, BCL‐2, and BAX protein expression in AAV‐NC and AAV‐DNMT1 testes. (l) Densitometric quantification of p‐AKT, BCL‐2, and BAX protein levels (p‐AKT normalized to total AKT). Data are presented as mean ± SEM. *p < 0.05, **p < 0.01, ***p < 0.001 compared with AAV‐NC group (Student's *t*‐test). *n* = 3 per group.

To further determine the impact of DNMT1 knockdown on LC senescence in vivo, we examined senescence‐associated phenotypes. SA‐β‐gal staining revealed a significantly increased number of senescent blue‐stained cells in the AAV‐DNMT1 group, predominantly localized in the testicular interstitium (Figure S5h,i). RT‐qPCR analysis showed that the expression of SASP factors (*Tnf*, *Il1b*, and *Il6*) was significantly elevated (Figure S5j). In addition, Western blot analysis confirmed that the protein level of the senescence marker p16 was markedly increased in the AAV‐DNMT1 group (Figure S5k,l). Collectively, these in vivo observations are fully consistent with our in vitro LC data, further verifying that DNMT1 deficiency accelerates LC senescence. Moreover, they confirm that DNMT1 depletion suppresses testicular testosterone synthesis through the FTO–HSP90AA1–AKT signaling cascade.

## 4 Discussion

Testosterone is essential for male sexual development and maintenance of reproductive function. Clinical evidence indicates that serum testosterone levels peak around the third decade of life and progressively decline thereafter (Rodrigues Dos Santos and Bhasin 2021; Zhu et al. 2022). Nowadays, testosterone replacement therapy (TRT) is currently the most widely applied intervention to alleviate hypogonadal symptoms and delay aging‐related functional decline; its efficacy remains suboptimal and is accompanied by notable contraindications and adverse effects, including polycythemia, cardiovascular events, and prostate‐related morbidity (Bal et al. 2026; Lundy et al. 2020; Neidhart et al. 2024; Zhao et al. 2023). Consequently, elucidating the mechanistic basis of age‐associated testosterone decline is of paramount importance for developing novel strategies aimed at enhancing endogenous steroidogenesis and identifying actionable therapeutic targets. In recent years, senescent cells have emerged as therapeutic targets in age‐related disease (Khalil et al. 2023). Leydig cells are among the earliest testicular cell types to experience senescence (Cui et al. 2025; Liu et al. 2025), and tailored efforts to increase endogenous testosterone output and postpone LC senescence are a promising therapeutic approach.

Epigenetic reprogramming is a hallmark of cellular senescence (López‐Otín et al. 2023). Among epigenetic modifications, DNA methylation plays a fundamental role in regulating gene transcription and maintaining cellular homeostasis. DNMT1, the principal maintenance methyltransferase, is responsible for preserving genomic DNA methylation patterns during replication. Emerging evidence has linked DNMT1 dysregulation to senescence and age‐related diseases (Camacho et al. 2025; Hahn et al. 2020; Patil et al. 2024); however, its specific role in testicular aging and LC‐mediated testosterone synthesis remains poorly understood. Elucidating the mechanistic involvement of DNMT1 function may provide a conceptual framework for improving age‐related testosterone synthesis dysfunction.

In the present study, we first systematically characterized the expression patterns of DNMT1 and testosterone in both aging rat testes and senescent primary LCs in vitro. Our data revealed that aging is consistently associated with reduced DNMT1 expression, diminished testosterone levels, and downregulated expression of key steroidogenic enzymes (CYP11A1, CYP17A1, and StAR). Through gain‐ and loss‐of‐function approaches, we demonstrated that DNMT1 knockdown significantly suppresses testosterone synthesis and key steroidogenic enzyme expression, whereas DNMT1 overexpression exerts reciprocal effects, establishing DNMT1 as a positive regulator of LC steroidogenic function. Similar to this, other studies have discovered that DNMT1 activity steadily declines with age (Li et al. 2010). Low‐dose radiation may hinder the production of testosterone by altering gene methylation through DNMT1 (Rolls et al. 2024). Moreover, we observed that DNMT1 knockdown induces LC senescence, whereas DNMT1 overexpression reverses the senescent phenotype and restores testosterone production in senescent LCs, suggesting that DNMT1 functions as a suppressor of LC senescence.

To dissect the molecular underpinnings of DNMT1‐mediated regulation of LC function, we performed RNA‐seq on DNMT1‐knockdown LCs and identified *Fto*, encoding the m^6^A demethylase FTO, as a significantly upregulated transcript. Integrated ChIP‐qPCR and MSP analyses confirmed that DNMT1 specifically binds to the CpG island within the *Fto* promoter and promotes its methylation. Functionally, DNMT1 knockdown increased, whereas DNMT1 overexpression decreased, FTO expression at both mRNA and protein levels. Notably, FTO overexpression did not significantly affect DNMT1 expression, positioning FTO as a downstream effector of DNMT1 rather than a reciprocal regulator.

FTO is a well‐characterized m^6^A demethylase that specifically erases m^6^A modifications from mRNA. By dynamically modulating RNA m^6^A levels, FTO regulates RNA stability, translation efficiency, and splicing, thereby participating in a broad spectrum of physiological and pathological processes (Li et al. 2022; Zuidhof and Calkhoven 2022). Research has demonstrated a positive correlation between the total sperm count, fertilization rate, embryo development rate, and the rate of high‐quality embryos and the amount of FTO mRNA in sperm (Pereira et al. 2021). On the other hand, other research has shown that aged mice's hearts, brains, lungs, and kidneys have higher levels of FTO expression (Krejčí et al. 2023). Through the JNK signaling pathway, IL‐17A increases FTO expression in human umbilical vein endothelial cells (HUVECs), causing endothelial senescence (Li, Luo, et al. 2023). However, the functional relevance of FTO in age‐related testosterone decline has remained unexplored. Here, we found that FTO expression is significantly upregulated in both aging rat testes and senescent LCs. Functional experiments confirmed that FTO overexpression markedly reduces testosterone production and downregulates steroidogenic enzyme expression. Mechanistically, catRAPID in silico prediction, combined with RIP‐qPCR validation, identified *Hsp90aa1* mRNA as a direct binding substrate of FTO. FTO knockdown and overexpression dynamically modulated the m^6^A modification status, mRNA abundance, and protein levels of HSP90AA1 in LCs. Importantly, DNMT1 knockdown did not alter the DNA methylation level of the *Hsp90aa1* promoter but significantly reduced HSP90AA1 expression, and concurrent FTO knockdown rescued the suppressive effect of DNMT1 deficiency on HSP90AA1. Collectively, these findings establish that FTO negatively regulates HSP90AA1 expression through m^6^A modification, whereas DNMT1 modulates HSP90AA1 indirectly via the FTO axis rather than through direct promoter DNA methylation.

HSP90AA1 is the gene encoding heat shock protein 90α (Hsp90α), which plays a crucial role in maintaining the stability of signaling proteins and promoting cell survival and growth (Hu et al. 2022; Yang et al. 2023). One of Hsp90α's primary customer proteins is AKT (Li, Wang, et al. 2023; Piredda et al. 2019). The AKT signaling pathway, a prototypical anti‐apoptotic cascade, has been implicated in testosterone secretion (Chen et al. 2020; Li, Yao, et al. 2023; Sun et al. 2022). We therefore hypothesized that HSP90AA1 promotes testosterone synthesis by activating AKT signaling, thereby inhibiting LC apoptosis and preserving LC viability. As predicted, HSP90AA1 overexpression significantly elevated testosterone levels and steroidogenic enzyme expression, increased p‐AKT and BCL‐2, and reduced BAX in LCs. Conversely, pharmacological inhibition of HSP90AA1 using 17‐AAG produced opposite effects. Notably, neither manipulation of HSP90AA1 expression altered DNMT1 or FTO levels, confirming that HSP90AA1 functions as a downstream effector of the DNMT1–FTO axis rather than a feedback regulator.

To rigorously establish the hierarchical architecture of the DNMT1–FTO–HSP90AA1–AKT axis, we performed a series of rescue experiments. FTO knockdown reversed the inhibitory effects of DNMT1 deficiency on HSP90AA1 expression, AKT phosphorylation, and testosterone production, whereas also attenuating LC senescence. HSP90AA1 overexpression rescued the functional impairments induced by FTO overexpression, whereas 17‐AAG treatment abrogated the protective effects of FTO knockdown. TUNEL staining corroborated these molecular findings, confirming that this axis exerts hierarchical control over LC apoptosis. Collectively, these multi‐layered rescue experiments definitively establish a linear DNMT1–FTO–HSP90AA1–AKT signaling cascade that modulates LC senescence and steroidogenic function.

Subsequently, we examined the dynamic alterations of the above molecular axis in testicular tissues of aging rats and senescent primary LCs in vitro. Our results confirmed that senescent LCs exhibit reduced DNMT1 expression, which diminishes *Fto* promoter DNA methylation and consequently upregulates FTO expression. Elevated FTO, in turn, decreases the m^6^A modification level of *Hsp90aa1* transcripts and suppresses HSP90AA1 expression, leading to markedly reduced AKT phosphorylation and impaired AKT‐mediated anti‐apoptotic signaling. This hierarchical cascade ultimately exacerbates LC apoptosis and senescence, culminating in diminished endogenous testosterone synthesis. To validate these findings in vivo, we established a testicular DNMT1‐knockdown rat model via local intratesticular injection of adeno‐associated virus, which recapitulated the aforementioned molecular and functional alterations. Collectively, this study elucidates the mechanism by which DNMT1 governs steroidogenic function in aging LCs. Mechanistically, DNMT1 alleviates LC senescence and, through FTO‐dependent m^6^A modification of *Hsp90aa1*, sustains HSP90AA1 expression to activate downstream AKT signaling, thereby inhibiting LC apoptosis and preserving testosterone synthesis (Figure 8).

**FIGURE 8:**
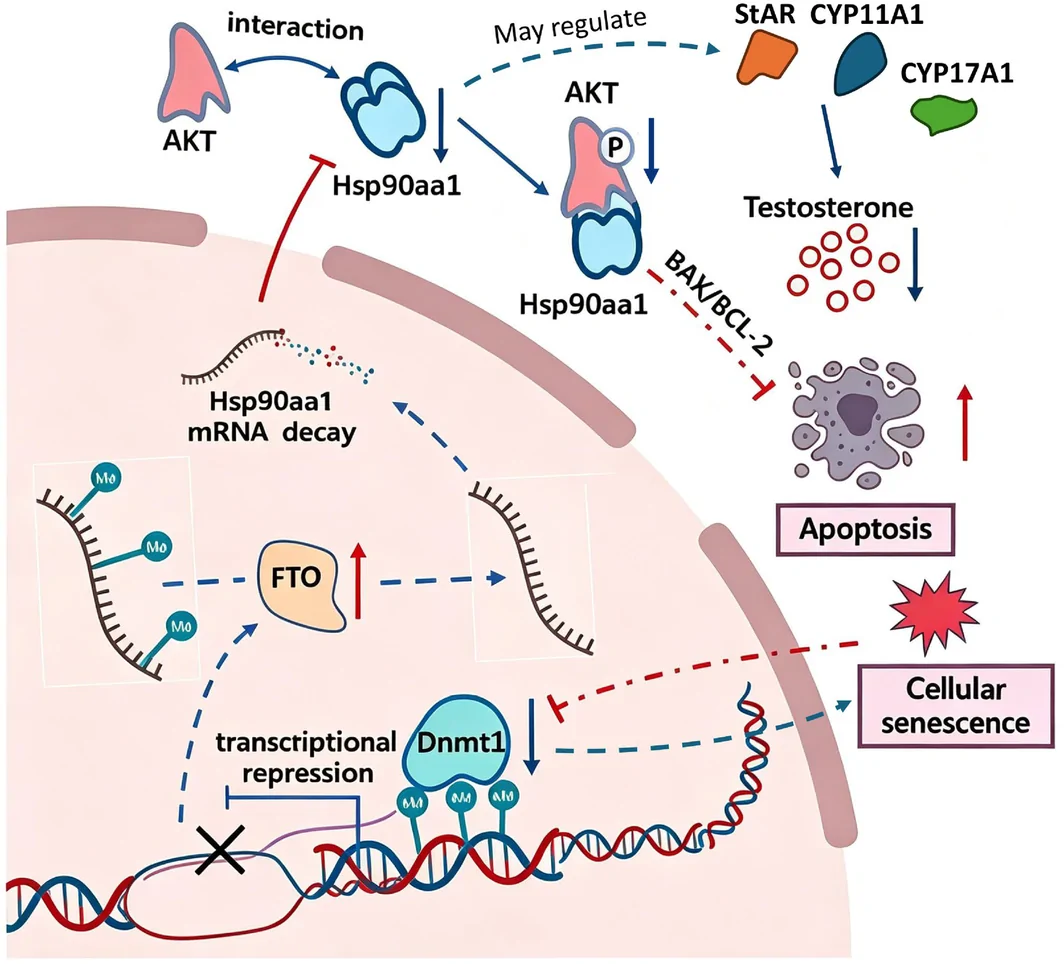
Schematic model of DNMT1‐mediated regulation of testosterone synthesis in Leydig cells. During LC senescence, DNMT1 expression declines. Reduced DNMT1 promotes LC senescence on one hand, and on the other hand leads to hypomethylation of the *Fto* promoter, thereby derepressing FTO transcription and protein expression. Elevated FTO, acting as an m^6^A demethylase, decreases the m^6^A modification level of *Hsp90aa1* transcripts, which suppresses HSP90AA1 expression. Downregulation of HSP90AA1 impairs AKT phosphorylation (p‐AKT) and blunts downstream anti‐apoptotic signaling, as evidenced by reduced BCL‐2 and increased BAX expression. This signaling cascade ultimately promotes LC apoptosis, culminating in diminished testosterone synthesis.

Taken together, our findings establish DNMT1 as a critical regulator of age‐related testosterone decline, positioning it as a promising therapeutic target for enhancing endogenous testosterone synthesis. Importantly, the DNMT1–FTO–HSP90AA1 axis offers a mechanistic alternative to exogenous testosterone replacement therapy (TRT), which is associated with limited efficacy and adverse effects. This axis holds considerable pharmacological potential, as selective small‐molecule inhibitors targeting DNMT1 and m^6^A modifiers provide feasible therapeutic strategies for mitigating male reproductive aging.

Several limitations of this study should be acknowledged. First, although MeRIP‐qPCR confirmed that FTO modulates the m^6^A modification of *Hsp90aa1* mRNA, the precise m^6^A residues on *Hsp90aa1* transcripts have not been mapped, and the functional contribution of individual modification sites remains undefined, precluding a more detailed mechanistic understanding of the DNMT1–FTO regulatory axis. Future m^6^A‐seq and site‐directed mutagenesis studies will address this gap. Second, our MSP assay lacked appropriate positive controls, including SssI‐treated fully methylated DNA and whole‐genome‐amplified unmethylated DNA, and the MSP products were not verified by Sanger sequencing. These limitations are partially compensated by the observation of single amplification products of the expected size and validation by ChIP‐qPCR; we will include these controls and perform Sanger sequencing in future studies. Third, our in vivo experiments were limited to single‐gene DNMT1 knockdown in rats; although AAV9 efficiently transduces LCs in mice (Xia et al. 2022; Zhang et al. 2024), its cell‐type specificity in rats requires further verification, and future dual‐gene rescue experiments will more rigorously establish the in vivo hierarchical relationships within this axis. Fourth, the DNMT1–FTO and HSP90AA1–AKT axes have not yet been validated in human testicular samples. Given the potential interspecies differences between rats and humans in LC senescence progression, hormonal regulatory networks, and gene expression profiles, validation in human samples is essential to confirm the translational relevance of our findings. Finally, while this study focused on FTO as the m^6^A effector mediating HSP90AA1 regulation, other key m^6^A modifiers—including METTL3 and ALKBH5—may also participate in this process. Their potential involvement was not examined here, and future studies will investigate their roles to comprehensively delineate the m^6^A regulatory network governing LC senescence and testosterone synthesis.

## 5 Conclusions

DNMT1 drives DNA methylation at the *Fto* promoter, thereby suppressing FTO expression, which in turn sustains HSP90AA1 expression by limiting FTO‐mediated m^6^A demethylation of *Hsp90aa1* transcripts. Elevated HSP90AA1 then activates AKT signaling, inhibits Leydig cell apoptosis, and ultimately promotes testosterone production. Collectively, these findings identify the DNMT1–FTO–HSP90AA1–AKT axis as a potential therapeutic target for enhancing endogenous testosterone synthesis during aging.

## Author Contributions

Yaqi Li and Yulei Duan: designed the project, performed the data analysis, wrote the manuscript, and generated research funds. Zihang Tian, Yibing Gao, Yufei Qiao, and Shuang Zhao: performed the experiments. Wenyan Li: provided technical help and helped draft the manuscript. Zheng Gao: supervised the research and helped draft the manuscript. Siyun Niu: generated research funds, conceived the work, and reviewed and edited the manuscript. All authors read and approved the final manuscript.

## Funding

This work was supported by the National Natural Science Foundation of China (82374180), S&T Program of Hebei (223777134D), Supported by Medical Science Foundation of Hebei University (2023B10), Hebei Key Laboratory of Aging and Health (SZX202431), President's Research Fund of Hebei University (XZJJ202506), and the Central Guidance for Local Science and Technology Development Fund Project of Hebei Province (264Z2504G).

## Conflicts of Interest

The authors declare no conflicts of interest.

## Supporting information


**Figure S1:** DNMT1 and testosterone levels are reduced in senescent Leydig cells in vitro. (a) Identification of primary Leydig cells by 3β‐HSD staining (positive cells appear blue). (b) Representative images of SA‐β‐gal staining in control and aged LCs. (c) Quantification of SA‐β‐gal‐positive cell rate. (d) RT‐qPCR analysis of SASP factors (*Tnf‐α*, *Il‐1β*, and *Il‐6*) and the senescence marker *p16* mRNA expression in aged LCs relative to controls. (e) Representative Western blot images of p16 protein expression. (f) Densitometric quantification of p16 Western blot bands. (g) Testosterone production in aged LCs measured by ELISA. (h) RT‐qPCR analysis of Cyp11a1, Cyp17a1, Star, and Dnmt1 mRNA expression. (i) Representative Western blot images of CYP11A1, CYP17A1, StAR, and DNMT1 protein expression. (j) Densitometric quantification of Western blot bands shown in (i). Data are presented as mean ± SEM. *p < 0.05, **p < 0.01, ***p < 0.001 compared with control group; unpaired two‐tailed Student's *t*‐test; *n* = 3 per group.
**Figure S2:** Pharmacological inhibition of HSP90AA1 suppresses AKT signaling and testosterone synthesis in Leydig cells. (a–c) Determination of optimal 17‐AAG intervention conditions. (a) CCK‐8 assay assessing cell viability after treatment with increasing concentrations of 17‐AAG (0, 0.5, 1, 2 μM) for 24, 48, or 72 h. (b) Representative Western blot images of total AKT and phosphorylated AKT (p‐AKT) expression following 17‐AAG treatment at indicated concentrations and durations. (c) Densitometric quantification of p‐AKT protein levels, compared to total AKT. (d) Testosterone levels in the culture supernatant measured by ELISA after 17‐AAG treatment (0.5 μM, 48 h). (e) Representative Western blot images showing downregulation of CYP11A1, CYP17A1, StAR, and DNMT1 protein expression following 17‐AAG treatment. (f) Densitometric quantification of CYP11A1, CYP17A1, and StAR protein levels. (g) Representative Western blot images showing that 17‐AAG treatment decreases p‐AKT and BCL‐2 expression while increasing BAX expression in LCs. (h) Densitometric quantification of p‐AKT, BCL‐2, and BAX protein levels. (i) Representative Western blot images showing that pharmacological inhibition of HSP90AA1 does not affect DNMT1 or FTO expression. (j) Densitometric quantification of DNMT1 and FTO protein levels. Data in (a–j) are presented as mean ± SEM. *p < 0.05, **p < 0.01, ***p < 0.001 compared with 0 μM or Control group. Student's *t*‐test for (d, f, h, j); ANOVA and Dunnett's T3 test for (a, c). *n* = 3 per group. (k) Representative Western blot images of p‐AKT, BCL‐2, and BAX expression in control, 17‐AAG, shFTO, and shFTO+17‐AAG groups. (l) Densitometric quantification of protein bands shown in (k). Data in (k, l) are presented as mean ± SEM. *p < 0.05, **p < 0.01, ***p < 0.001 compared with indicated groups (ANOVA and Tukey test). *n* = 3 per group.
**Figure S3:** Age‐related alterations of the FTO–HSP90AA1 axis in senescent Leydig cells in vitro. (a) MSP analysis of *Fto* promoter methylation status; M: methylated; U: unmethylated. (b) MSP statistical results. (c, d) RT‐qPCR (c) and Western blot (d) analysis showing upregulation of FTO expression in aged LCs compared with controls. (e) Densitometric quantification of FTO protein levels. (f) Global cellular m^6^A levels in control and aged LCs. (g) MeRIP‐qPCR analysis of m^6^A modification levels of *Hsp90aa1* transcripts in control and aged LCs. (h, i) RT‐qPCR (h) and Western blot (i) analysis demonstrating downregulation of *Hsp90aa1* mRNA and HSP90AA1 protein expression in aged LCs. (j) Densitometric quantification of HSP90AA1 protein levels. Data are presented as mean ± SD. *p < 0.05, **p < 0.01, ***p < 0.001 compared with Control group (Student's *t*‐test). *n* = 3 per group.
**Figure S4:** Expression alterations of the DNMT1–FTO–HSP90AA1–AKT axis in primary Leydig cells isolated from young and aged rats. (a) Identification of primary LCs by 3β‐HSD staining (positive cells appear blue). (b) Representative images of SA‐β‐gal staining in primary LCs isolated from 2‐month‐old (young) and 18‐month‐old (aged) rats (scale bar = 200 μm). (c) Quantification of SA‐β‐gal‐positive cells, expressed as percentage of total cells. (d) Testosterone levels in the culture supernatant of primary LCs from young and aged rats, measured by ELISA. (e, f) MSP showing reduced *Fto* promoter methylation in aged LCs. (g) MeRIP‐qPCR revealing reduced m^6^A modification of *Hsp90aa1* transcripts in aged LCs. (h, i) RT‐qPCR (h) and Western Blot (i) analysis showing decreased DNMT1 and HSP90AA1 and increased FTO at both mRNA and protein levels in aged LCs. (j) Densitometric quantification of DNMT1, FTO, and HSP90AA1 protein levels. (k) Representative Western blot images of p‐AKT, total AKT, BCL‐2, and BAX expression in primary LCs from young and aged rats. (l) Densitometric quantification of p‐AKT, BCL‐2, and BAX protein levels (p‐AKT normalized to total AKT). Data are presented as mean ± SEM. *p < 0.05, **p < 0.01, ***p < 0.001 compared with 2 months (Student's *t*‐test). *n* = 3 per group.
**Figure S5:** Optimization of in vivo DNMT1 knockdown dosage and verification of DNMT1 deficiency‐induced testicular senescence. (a) Representative images of testicular morphology from AAV‐NC and AAV‐DNMT1 groups at indicated viral titers (low, medium, high). (b) Statistical analysis of testicular index (testis weight/body weight ratio) in each group. (c, d) RT‐qPCR (c) and Western blot (d) analysis showing that the high‐dose AAV‐DNMT1 achieves the most efficient DNMT1 knockdown in testicular tissues. (e) Densitometric quantification of DNMT1 protein levels. (f) Representative immunohistochemical staining images of DNMT1 in testicular sections from AAV‐NC and AAV‐DNMT1 groups (high titer) (scale bar = 50 μm). (f, g) IHC confirming reduced DNMT1 expression in the high‐titer group. (h, i) SA‐β‐gal staining showing increased senescence in DNMT1‐deficient testes. (j) RT‐qPCR analysis of SASP factors (*Tnf‐α*, *Il‐1β*, and *Il‐6*) mRNA expression in AAV‐NC and AAV‐DNMT1 testes. (k) Representative Western blot images of p16 protein expression. (l) Densitometric quantification of p16 protein levels. Data are presented as mean ± SEM. *p < 0.05, **p < 0.01, ***p < 0.001 compared with AAV‐NC group. ANOVA and Dunnett's T3 test for (b, c, e); Student's *t*‐test for (g, i, j, l). *n* = 3 per group.

## Data Availability

The data that support the findings of this study are available from the corresponding author upon reasonable request.
