# Supplementary material for: DNMT1 Regulates Testosterone Synthesis in Aged Rat Leydig Cells via FTO‐Mediated HSP90AA1 m6A Levels
Source: Aging Cell. 2026 Oct 12;25(10):e70760. doi: 10.1111/acel.70760 (PMC13658364; doi:10.1111/acel.70760)
Supplement: Supplementary file 1 [file ACEL-25-e70760-s001.pdf]

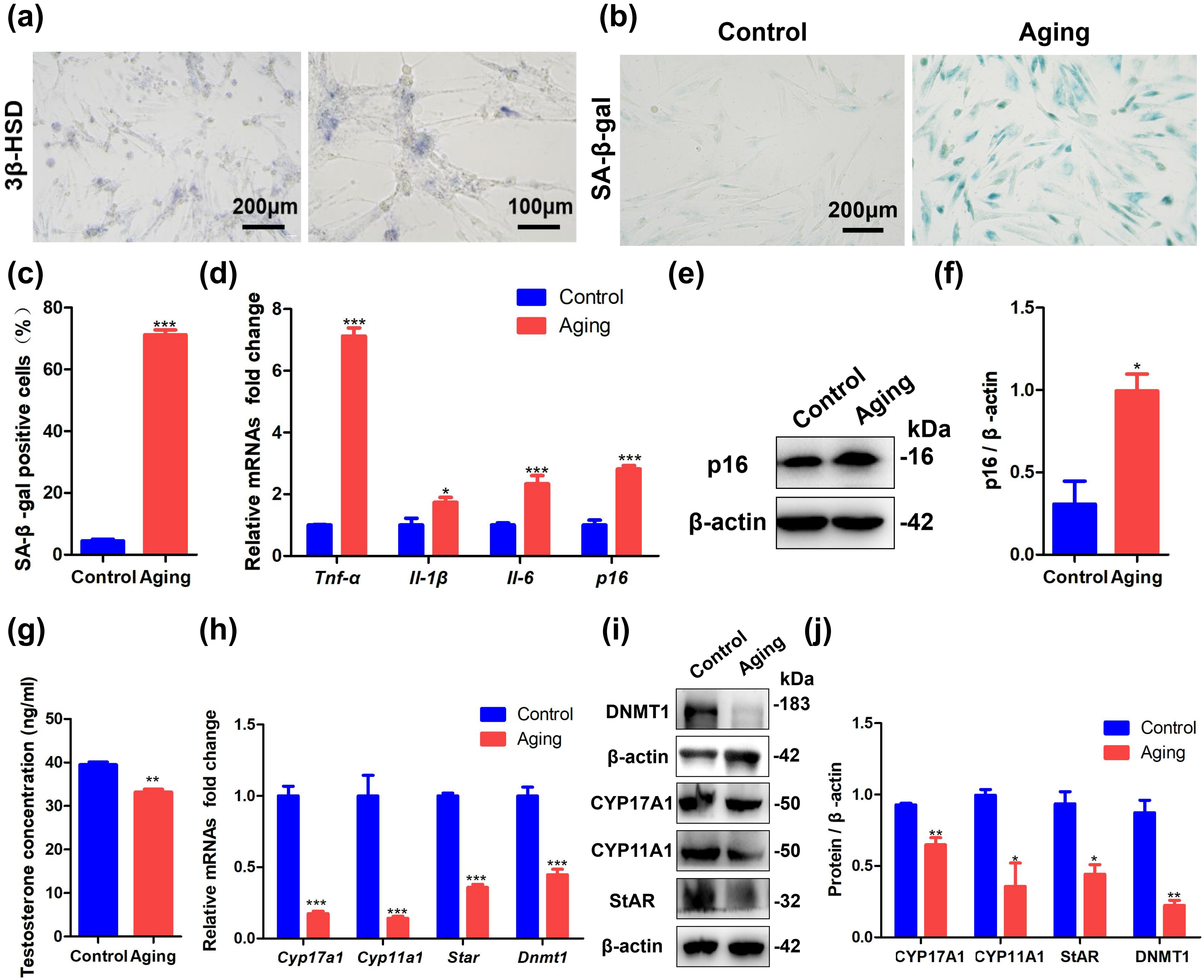

(a)
3β-HSD
200μm
100μm
(b)
Control
Aging
SA-β-gal
200μm
(c)
SA-β -gal positive cells（%）
Control
Aging
(d)
Relative mRNAs fold change
Control
Aging
Tnf-α
Il-1β
Il-6
p16
(e)
Control
Aging
kDa
p16
-16
β-actin
-42
(f)
p16 / β -actin
Control
Aging
(g)
Testosterone concentration (ng/ml)
Control
Aging
(h)
Relative mRNAs fold change
Control
Aging
Cyp17a1
Cyp11a1
Star
Dnmt1
(i)
Control
Aging
kDa
DNMT1
-183
β-actin
-42
CYP17A1
-50
CYP11A1
-50
StAR
-32
β-actin
-42
(j)
Protein / β -actin
Control
Aging
CYP17A1
CYP11A1
StAR
DNMT1

Supplementary Figure 1. DNMT1 and testosterone levels are reduced in senescent Leydig cells *in vitro*. (a) Identification of primary Leydig cells by 3β-HSD staining (positive cells appear blue). (b) Representative images of SA-β-gal staining in control and aged LCs. (c) Quantification of SA-β-gal-positive cell rate. (d) RT–qPCR analysis of SASP factors (*Tnf-α*, *Il-1β*, and *Il-6*) and the senescence marker *p16* mRNA expression in aged LCs relative to controls. (e) Representative Western blot images of p16 protein expression. (f) Densitometric quantification of p16 Western blot bands. (g) Testosterone production in aged LCs measured by ELISA. (h) RT–qPCR analysis of Cyp11a1, Cyp17a1, Star, and Dnmt1 mRNA expression. (i) Representative Western blot images of CYP11A1, CYP17A1, StAR, and DNMT1 protein expression. (j) Densitometric quantification of Western blot bands shown in (i). Data are presented as mean ± SEM. *$p < 0.05$, **$p < 0.01$, ***$p < 0.001$ compared with control group; unpaired two-tailed Student's t-test; n=3 per group.

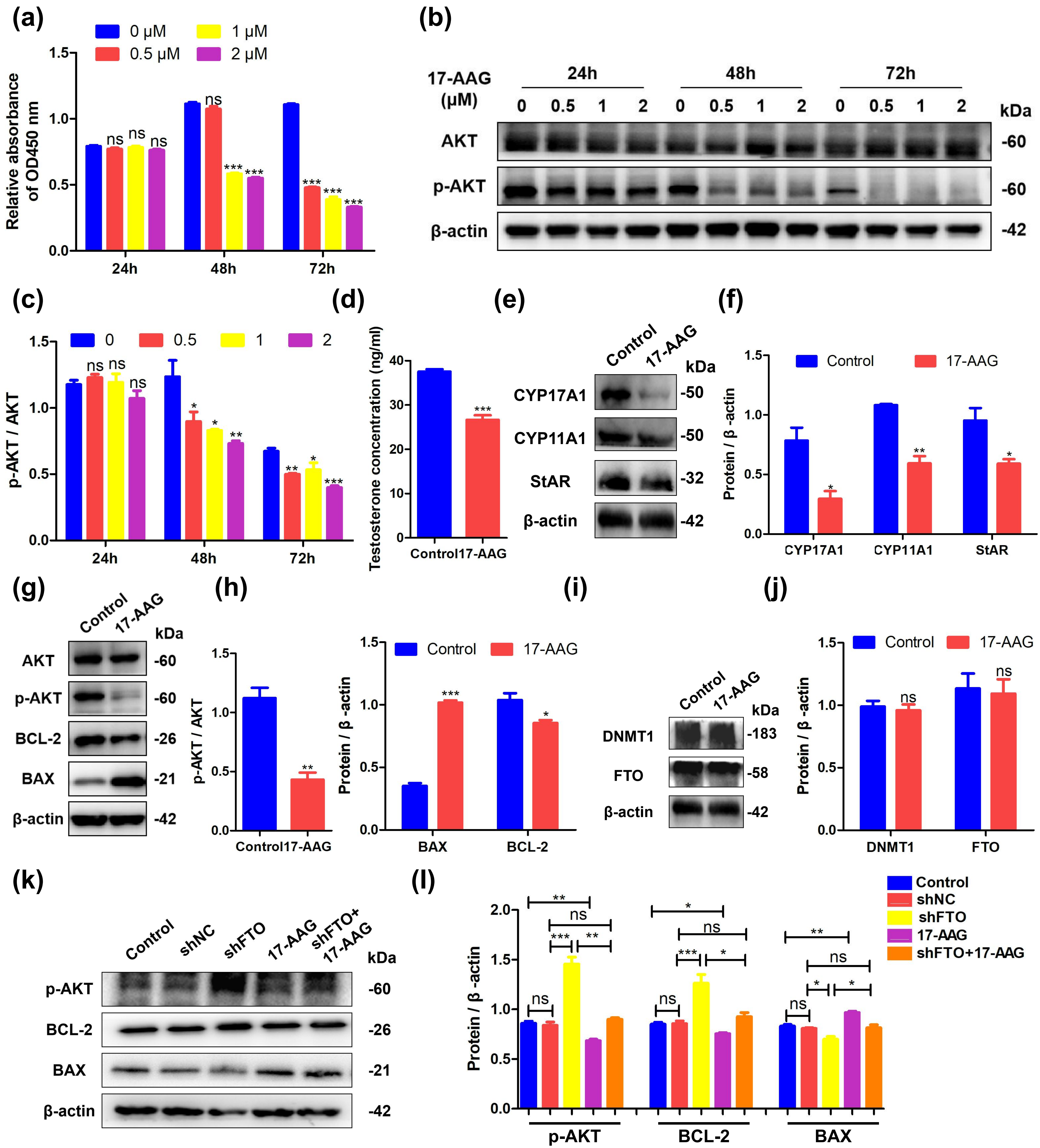
(a)
0 μM
0.5 μM
1 μM
2 μM
Relative absorbance of OD450 nm
24h
48h
72h
ns
***
(b)
17-AAG (μM)
24h
48h
72h
0 0.5 1 2 0 0.5 1 2 0 0.5 1 2
kDa
AKT
p-AKT
β-actin
-60
-60
-42
(c)
0
0.5
1
2
p-AKT / AKT
24h
48h
72h
(d)
Testosterone concentration (ng/ml)
Control
17-AAG
(e)
Control
17-AAG
kDa
CYP17A1
CYP11A1
StAR
β-actin
-50
-50
-32
-42
(f)
Control
17-AAG
Protein / β -actin
CYP17A1
CYP11A1
StAR
(g)
Control
17-AAG
kDa
AKT
p-AKT
BCL-2
BAX
β-actin
-60
-60
-26
-21
-42
(h)
p-AKT / AKT
Control
17-AAG
Protein / β -actin
BAX
BCL-2
(i)
Control
17-AAG
kDa
DNMT1
FTO
β-actin
-183
-58
-42
(j)
Control
17-AAG
Protein / β -actin
DNMT1
FTO
(k)
Control
shNC
shFTO
17-AAG
shFTO+ 17-AAG
kDa
p-AKT
BCL-2
BAX
β-actin
-60
-26
-21
-42
(l)
Control
shNC
shFTO
17-AAG
shFTO+17-AAG
Protein / β -actin
p-AKT
BCL-2
BAX

Supplementary Figure S2. Pharmacological inhibition of HSP90AA1 suppresses AKT signaling and testosterone synthesis in Leydig cells. (a–c) Determination of optimal 17-AAG intervention conditions. (a) CCK-8 assay assessing cell viability after treatment with increasing concentrations of 17-AAG (0, 0.5, 1, 2 μM) for 24, 48, or 72 h. (b) Representative Western blot images of total AKT and phosphorylated AKT (p-AKT) expression following 17-AAG treatment at indicated concentrations and durations. (c) Densitometric quantification of p-AKT protein levels, compared to total AKT. (d) Testosterone levels in the culture supernatant measured by ELISA after 17-AAG treatment (0.5 μM, 48 h). (e) Representative Western blot images showing downregulation of CYP11A1, CYP17A1, StAR, and DNMT1 protein expression following 17-AAG treatment. (f) Densitometric quantification of CYP11A1, CYP17A1, and StAR protein levels. (g) Representative Western blot images showing that 17-AAG treatment decreases p-AKT and BCL-2 expression while increasing BAX expression in LCs. (h) Densitometric quantification of p-AKT, BCL-2, and BAX protein levels. (i) Representative Western blot images showing that pharmacological inhibition of HSP90AA1 does not affect DNMT1 or FTO expression. (j) Densitometric quantification of DNMT1 and FTO protein levels. Data in (a–j) are presented as mean ± SEM. $^*p < 0.05$, $^{**}p < 0.01$, $^{***}p < 0.001$ compared with 0 μM or Control group. Student's t-test for (d, f, h, j); ANOVA and Dunnett's T3 test for (a, c). n=3 per group. (k) Representative Western blot images of p-AKT, BCL-2, and BAX expression in control, 17-AAG, shFTO, and shFTO+17-AAG groups. (l) Densitometric quantification of protein bands shown in (k). Data in (k, l) are presented as mean ± SEM. $^*p < 0.05$, $^{**}p < 0.01$, $^{***}p < 0.001$ compared with indicated groups (ANOVA and Tukey test). n=3 per group.

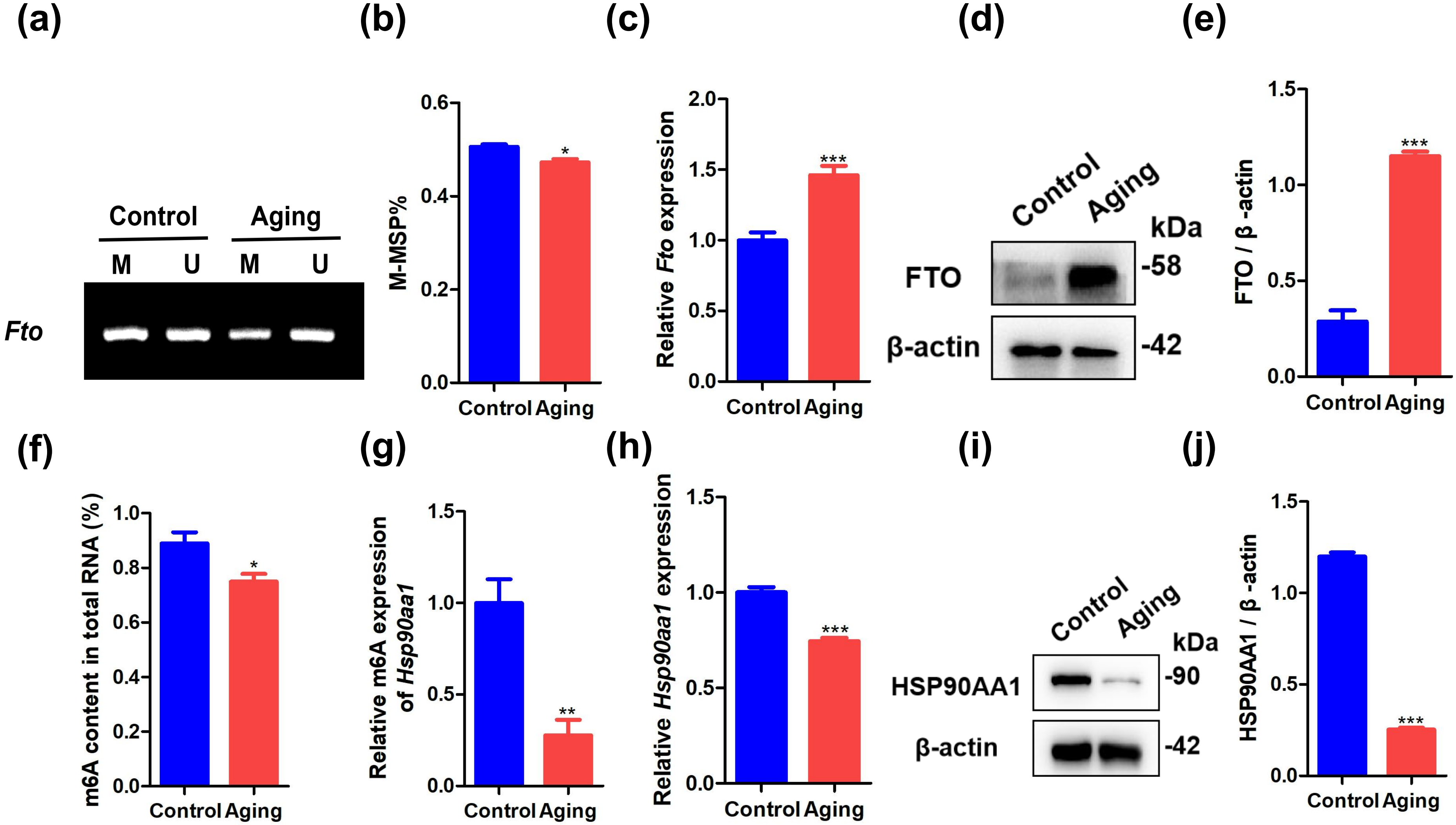
(a)
Control
Aging
M
U
M
U
Fto
(b)
M-MSP%
0.6
0.4
0.2
0.0
*
Control
Aging
(c)
Relative Fto expression
2.0
1.5
1.0
0.5
0.0
***
Control
Aging
(d)
Control
Aging
kDa
FTO
-58
β-actin
-42
(e)
FTO / β -actin
1.5
1.0
0.5
0.0
***
Control
Aging
(f)
m6A content in total RNA (%)
1.0
0.8
0.6
0.4
0.2
0.0
*
Control
Aging
(g)
Relative m6A expression of Hsp90aa1
1.5
1.0
0.5
0.0
**
Control
Aging
(h)
Relative Hsp90aa1 expression
1.5
1.0
0.5
0.0
***
Control
Aging
(i)
Control
Aging
kDa
HSP90AA1
-90
β-actin
-42
(j)
HSP90AA1 / β -actin
1.5
1.0
0.5
0.0
***
Control
Aging

Supplementary Figure S3. Age-related alterations of the FTO–HSP90AA1 axis in senescent Leydig cells *in vitro*. (a) MSP analysis of *Fto* promoter methylation status; M: methylated; U: unmethylated. (b) MSP statistical results. (c, d) RT–qPCR (c) and Western blot (d) analysis showing upregulation of FTO expression in aged LCs compared with controls. (e) Densitometric quantification of FTO protein levels. (f) Global cellular m6A levels in control and aged LCs. (g) MeRIP–qPCR analysis of m6A modification levels of *Hsp90aa1* transcripts in control and aged LCs. (h, i) RT–qPCR (h) and Western blot (i) analysis demonstrating downregulation of *Hsp90aa1* mRNA and HSP90AA1 protein expression in aged LCs. (j) Densitometric quantification of HSP90AA1 protein levels. Data are presented as mean ± SD. * $p < 0.05$, ** $p < 0.01$, *** $p < 0.001$ compared with Control group (Student's t-test). n=3 per group.

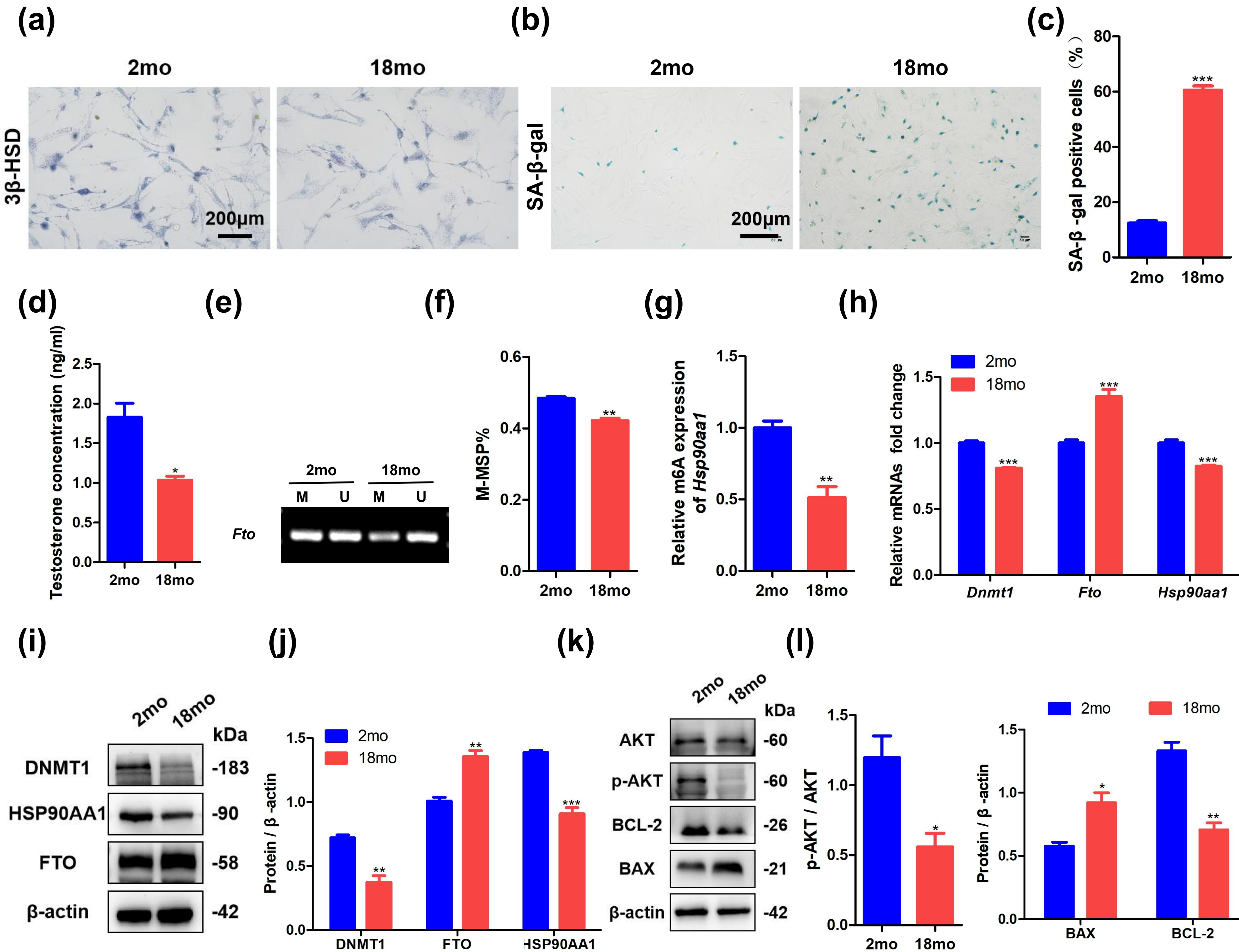

(a)
2mo
18mo
3β-HSD
200μm
(b)
2mo
18mo
SA-β-gal
200μm
(c)
SA-β -gal positive cells （%）
2mo
18mo
(d)
Testosterone concentration (ng/ml)
2mo
18mo
(e)
2mo
18mo
M U M U
Fto
(f)
M-MSP%
2mo
18mo
(g)
Relative m6A expression of Hsp90aa1
2mo
18mo
(h)
Relative mRNAs fold change
2mo
18mo
Dnmt1
Fto
Hsp90aa1
(i)
2mo
18mo
kDa
DNMT1 -183
HSP90AA1 -90
FTO -58
β-actin -42
(j)
Protein / β -actin
2mo
18mo
DNMT1
FTO
HSP90AA1
(k)
2mo
18mo
kDa
AKT -60
p-AKT -60
BCL-2 -26
BAX -21
β-actin -42
(l)
p-AKT / AKT
2mo
18mo
Protein / β -actin
BAX
BCL-2

Supplementary Figure S4. Expression alterations of the DNMT1–FTO–HSP90AA1-AKT axis in primary Leydig cells isolated from young and aged rats. (a) Identification of primary LCs by 3β-HSD staining (positive cells appear blue). (b) Representative images of SA-β-gal staining in primary LCs isolated from 2-month-old (young) and 18-month-old (aged) rats (scale bar = 200 μm). (c) Quantification of SA-β-gal-positive cells, expressed as percentage of total cells. (d) Testosterone levels in the culture supernatant of primary LCs from young and aged rats, measured by ELISA. (e, f) MSP showing reduced *Fto* promoter methylation in aged LCs. (g) MeRIP–qPCR revealing reduced m6A modification of *Hsp90aa1* transcripts in aged LCs. (h, i) RT–qPCR (h) and Western Blot (i) analysis showing decreased DNMT1 and HSP90AA1 and increased FTO at both mRNA and protein levels in aged LCs. (j) Densitometric quantification of DNMT1, FTO, and HSP90AA1 protein levels. (k) Representative Western blot images of p-AKT, total AKT, BCL-2, and BAX expression in primary LCs from young and aged rats. (l) Densitometric quantification of p-AKT, BCL-2, and BAX protein levels (p-AKT normalized to total AKT). Data are presented as mean ± SEM. * $p < 0.05$, ** $p < 0.01$, *** $p < 0.001$ compared with 2mo (Student's t-test). n=3 per group.

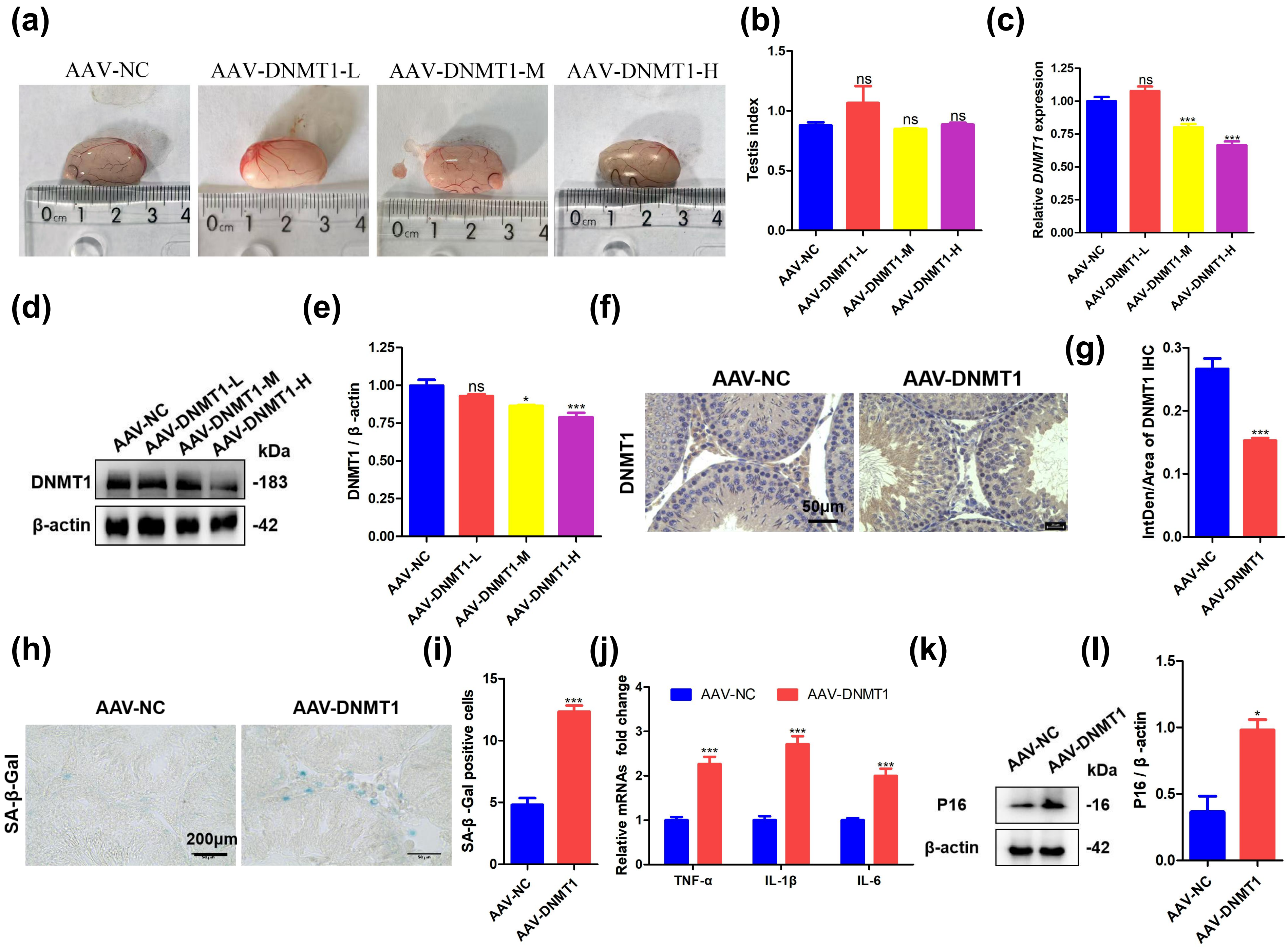

(a)
AAV-NC
AAV-DNMT1-L
AAV-DNMT1-M
AAV-DNMT1-H
(b)
Testis index
ns
ns
ns
AAV-NC
AAV-DNMT1-L
AAV-DNMT1-M
AAV-DNMT1-H
(c)
Relative DNMT1 expression
ns
***
***
(d)
AAV-NC
AAV-DNMT1-L
AAV-DNMT1-M
AAV-DNMT1-H
kDa
DNMT1
-183
β-actin
-42
(e)
DNMT1 / β -actin
ns
*
***
(f)
AAV-NC
AAV-DNMT1
DNMT1
50μm
(g)
IntDen/Area of DNMT1 IHC
***
(h)
AAV-NC
AAV-DNMT1
SA-β-Gal
200μm
(i)
SA-β -Gal positive cells
***
(j)
AAV-NC
AAV-DNMT1
Relative mRNAs fold change
***
***
***
TNF-α
IL-1β
IL-6
(k)
AAV-NC
AAV-DNMT1
kDa
P16
-16
β-actin
-42
(l)
P16 / β -actin
*

Supplementary Figure S5. Optimization of *in vivo* DNMT1 knockdown dosage and verification of DNMT1 deficiency-induced testicular senescence. (a) Representative images of testicular morphology from AAV-NC and AAV-DNMT1 groups at indicated viral titers (low, medium, high). (b) Statistical analysis of testicular index (testis weight/body weight ratio) in each group. (c, d) RT–qPCR (c) and Western blot (d) analysis showing that the high-dose AAV-DNMT1 achieves the most efficient DNMT1 knockdown in testicular tissues. (e) Densitometric quantification of DNMT1 protein levels. (f) Representative immunohistochemical staining images of DNMT1 in testicular sections from AAV-NC and AAV-DNMT1 groups (high titer) (scale bar = 50 μm). (f, g) IHC confirming reduced DNMT1 expression in the high-titer group. (h, i) SA-β-gal staining showing increased senescence in DNMT1-deficient testes. (j) RT-qPCR analysis of SASP factors (*Tnf-α*, *Il-1β*, and *Il-6*) mRNA expression in AAV-NC and AAV-DNMT1 testes. (k) Representative Western blot images of p16 protein expression. (l) Densitometric quantification of p16 protein levels. Data are presented as mean ± SEM. * $p < 0.05$, ** $p < 0.01$, *** $p < 0.001$ compared with AAV-NC group. ANOVA and Dunnett's T3 test for (b, c, e); Student's *t*-test for (g, i, j, l). n=3 per group.
